# Reliability of Difference Scores Obtained From Nested Data Within a Multivariate Generalizability Theory Framework

**DOI:** 10.1177/00131644261451746

**Published:** 2026-07-07

**Authors:** Rabia Karatoprak Ersen, Won-Chan Lee, Donald B. Yarbrough

**Affiliations:** 1 GESIS—Leibniz Institute for the Social Sciences, Cologne, Germany; 2 The University of Iowa, Iowa City, USA

**Keywords:** difference scores, multilevel data, multivariate generalizability theory, reliability, dependability, educational standards

## Abstract

The purpose of this study is to examine the reliability and dependability of difference scores computed as the change between a pretest and a posttest administered to assess the effectiveness of an intervention. The data-collection design involved a nested structure, with persons (*p*) nested within groups (*g*), and groups nested within sites (*s*). Multivariate generalizability theory was employed to estimate the reliability and dependability of difference scores at the levels of persons, groups, and sites. The *G* study designs included $\left(p^{•}:g^{•}:s^{•}\right)\;x\;i^{•}$ , $\left(p^{•}:s^{•}\right)\;x\;i^{•}$ , $\left(p^{•}:g^{•}\right)\;x\;i^{•}$ , $g^{•}\;x\;i^{•}$ , $p^{•}x\;i^{•}$ , and $s^{•}x\;i^{•}$ , with pretest and posttest serving as the two levels of the multivariate facet. In the designs with sites as the object of measurement, $\left(P^{•}:G^{•}:s^{•}\right)\;x\;I^{•}$ , $\left(P^{•}:s^{•}\right)\;x\;I^{•}$ , and $s^{•}x\;I^{•}$ , omitting groups within sites or persons within groups led to an underestimation of error variances and inflated generalizability and dependability coefficients. The relative error correlations increased, and the absolute error and universe score correlations decreased across $s^{•}x\;I^{•}$ , $\left(P^{•}:s^{•}\right)\;x\;I^{•}$ , and $\left(P^{•}:G^{•}:s^{•}\right)\;x\;I^{•}$ . Across all designs, generalizability and dependability coefficients were similar in magnitude, primarily due to the relatively small variance in items. Compared across different objects of measurement, both the generalizability and dependability coefficients were highest when the object of measurement was persons, and lowest when it was sites.

In practice, difference scores are utilized for various purposes under longitudinal or repeated-measures designs. For instance, experimental research designs are conducted to assess the effectiveness of new instructional methods, educational programs, or treatments in terms of changes in educational or psychological constructs. These situations involve administering pretests and posttests in multiple schools. Another example of interest concerns assessing changes in academic success. For instance, the Every Student Succeeds Act requires states to measure academic progress in elementary and middle schools. Thus, schools are responsible for demonstrating that they provide high-quality education. One of the indicators is academic progress. Administrators want to know how much academic progress students, classes, and thus schools made between different grades. In these cases, inferences can be made using difference scores, which are computed by subtracting pretest scores from posttest scores. The data from which the difference scores are obtained have a nested structure, such that students are nested within classes, and classes are nested within schools.

The Standards for Educational and Psychological Testing (American Educational Research Association et al., 2014) state that whenever the difference between observed scores is interpreted and used for actions, the reliability of difference scores needs to be reported. Moreover, if there is an intent or need to make interpretations at the group level, then the reliability of group means also needs to be reported. When difference scores are computed at the group level, the reliability of the group mean difference scores must be reported. Generalizability theory (G theory) offers comprehensive tools for estimating reliability, including the reliability of difference scores and group scores, while considering various sources of measurement error (Brennan, 2001a).

The reliability of school means using univariate G theory was examined, and it was found that excluding class-level variance could introduce bias into the reliability estimates (Wei & Haertel, 2011). The reliability of group mean difference scores has been examined in multiple studies using classical test theory (CTT) or G theory (e.g., Brennan, 1995; Brennan et al., 2003; Cronbach et al., 1997). However, the data in these studies did not encapsulate the nested structure of schools or did not include items as a source of error, which changes the G theory study design and definitions of error variances. Moreover, the effect of ignoring lower-level variances on the reliability of school or class mean difference scores has not been investigated. Reliability of difference scores requires consideration of correlated error between the pretest and posttest from which the difference scores are computed. Multivariate G theory is pertinent in such cases, enabling the explicit modeling of variance and covariance components in the computation of correlated errors. Furthermore, the variation in the object of measurement within a single dataset was not studied, and the importance of incorporating nesting when calculating the reliability of scores derived from a specific object of measurement was overlooked. The purpose of this study is to fill this gap by identifying the sources of error, examining their contribution to the reliability coefficients, and investigating how these coefficients change when the object of measurement varies in a nested design.

In doing so, this study makes three interrelated contributions: It extends the literature on the reliability of difference scores by applying a multivariate G theory approach to two-level nested pretest–posttest designs, which is a data structure typical of K-12, higher education, and large-scale assessment contexts that has not been fully represented in prior studies; it provides empirical evidence of how omitting nested facets inflates reliability coefficients and alters error correlations across designs; and it offers a step-by-step illustration of the analysis procedure that researchers and practitioners can follow when working with similarly structured data.

For this purpose, this study used an empirical data set consisting of pretest and posttest data administered to assess the effectiveness of a statewide intervention (Wade & Yarbrough, 2007). Teams of teachers from various school districts implemented the intervention for students. Within each school district, multiple teachers implemented the intervention, and each teacher worked with multiple students. Thus, the data had a nested structure such that students were nested within teachers, and teachers were nested within school districts. In this study, the terms *persons (p)*, *groups (g)*, and *sites (s)* are used to refer to students, teachers, and school districts, respectively. Prior research on the reliability of group scores often examines students nested within classes and classes nested within schools. However, in this study, groups are equivalent to classes (one level of nesting), and sites are equivalent to schools (two levels of nesting). With this framework, this study aims to answer the following research questions:

**Research Question 1 (RQ1):** How does the reliability of difference scores compare when the objects of measurement are (a) sites, (b) groups, or (c) persons?
**Research Question 2 (RQ2):** What are the impacts of neglecting nested facets, such as persons or groups, on the reliability of difference scores?

The remainder of this paper begins with a brief overview of CTT and G theory, followed by a review of previous research on difference scores. Then, the study design and analysis procedures are described. It concludes with a discussion of results in relation to the research questions.

## CTT, Univariate G Theory, and Multivariate G Theory

Suppose individuals respond to a set of essay prompts, and raters evaluate their responses. In this assessment scenario, essay prompts and raters are key sources of measurement error that should be incorporated into reliability estimation. CTT-based reliability-estimation methods treat error as a single undifferentiated entity and cannot separate multiple sources of error in the estimation process. However, G theory offers methodologies capable of accounting for different sources of error, allowing for the examination of their individual contributions to reliability.

Furthermore, G theory explicitly distinguishes between two types of error: relative error ( δ ) and absolute error ( Δ ). Relative error variance ( $\sigma^{2}\left(\delta \right)$ ) is used to estimate the generalizability coefficient ( $E\rho^{2}$ ), which serves as an index for norm-referenced interpretations. Absolute error variance ( $\sigma^{2}\left(\Delta \right)$ ) is used to estimate the dependability coefficient ( Φ ), which is an index for domain-referenced inferences. These error types also serve as the basis for other indices such as the signal–noise ratio (S/N; Brennan & Kane, 1977) and the error–tolerance ratio (E/T; Kane, 1996). In contrast, reliability indices in CTT involve relative error variance, making them more suitable for norm-referenced interpretations. CTT typically does not distinguish between absolute and relative error.

*G* theory constitutes both univariate and multivariate methodologies. One of the distinct advantages of multivariate G theory over univariate G theory is its ability to account for correlated error variances and to model composite scores. For instance, difference scores, such as the difference between posttest and pretest scores, can be specified as composite scores. In this framework, both variance and covariance components for pretest and posttest scores can be estimated and used in calculating reliability coefficients for difference scores. However, the univariate G theory approach lacks the capacity to model difference scores as composites or to estimate covariance between pretest and posttest scores. Typically, CTT assumes zero covariance between errors of pretest and posttest scores. Multivariate G theory relaxes these assumptions, allowing for a more accurate and flexible estimation of the reliability of difference scores.

## Research on Difference Scores

Researchers often avoid using difference scores mainly for several reasons, as outlined by Gu et al. (2018). First, difference scores tend to have low reliability, implying a weak correlation between observed and true difference scores. Second, the reliability of difference scores is generally lower than that of the test scores from which the difference scores are obtained. Third, when reliability is low, the measurement precision of difference scores at the individual level becomes limited. Fourth, it is not possible to observe both a high correlation between pretest and posttest scores and a high reliability of difference scores simultaneously. Finally, difference scores can be potentially defective given that the correlation between difference scores and pretest scores can be negative, raising concerns about their interpretability.

However, studies have provided evidence contradicting the notion that psychometric properties of difference scores are inadequate for making valid inferences. Thomas and Zumbo (2011) demonstrated the valid use of difference scores in repeated-measures experimental designs. Gu et al. (2018) provided analytical explanations based on CTT by differentiating individual and group-level change scores. Moreover, difference scores can be useful if indices for reliability are selected according to the intended interpretations, which may be norm-referenced or domain-referenced (Yin & Brennan, 2002). G theory can provide $E{\hat{\rho }}^{2}$ , $\hat{\Phi }$ , S/N, or E/T for both norm- and domain-referenced interpretations (Brennan et al., 2003; Brennan & Kane, 1977; Kane, 1996; Miller & Kane, 2001).

A key determinant for which reliability index should be used is the intended interpretations of difference scores. If the focus is not on ranking individuals based on their difference scores, the appropriate index to use is a coefficient representing the reliability of absolute change, for which G theory is particularly well suited (Yin & Brennan, 2002). Miller and Kane (2001) proposed using E/T, which can be computed using ${\hat{\sigma }}^{2}\left(\delta \right)$ or ${\hat{\sigma }}^{2}\left(\Delta \right)$ for norm-referenced or domain-referenced interpretations. Brennan et al. (2003) examined the reliability of group mean difference scores using both $E{\hat{\rho }}^{2}$ and $\hat{\Phi }$ , as well as the E/T ratio. Their analysis, conducted using multivariate G theory, revealed that both relative-type and absolute-type errors were correlated in their data.

Existing studies using the G theory framework differ in terms of their *G* study designs. Yin and Brennan (2002) did not strictly adhere to G theory conventions, but their design can be reasonably interpreted as a pxi design. The study design of Miller and Kane (2001) was px(i:c) , where persons were crossed with items, and items were nested within categories. Brennan et al. (2003) used p:(sxc) where persons were nested within school districts, and districts were crossed with cohorts. In their multivariate G theory analysis, this design was extended to $p^{•}:(s^{•}xc^{•})$ , where the object of measurement was groups rather than individuals. The inclusion of cohorts served to replicate the measurement.

When estimating the reliability of difference scores for groups, the nested structure of the data is a source of variance. In Yin and Brennan (2002), difference scores for sites (i.e., school districts) were examined using CTT. In Brennan et al. (2003), difference scores were expressed in grade equivalents for a site, using a p:(sxc) and $p^{•}:\left(s^{•}xc^{•}\right)$ design. However, despite the nested nature of the data, where persons are within groups and groups within sites, this nesting was not incorporated into the reliability analysis. As Wei and Haertel (2011) noted, ignoring group-level variance can introduce bias in the generalizability coefficient for site scores. Furthermore, the inclusion of the item facet in the reliability analysis enables discussion of both generalizability and the dependability coefficient. The purpose of this study is to address these gaps by examining both generalizability and dependability coefficients of difference scores obtained from two-level nested data across multivariate G theory designs varying in object of measurement and inclusion of nesting levels. Unlike prior studies that did not fully represent the nested structure of the data, this study jointly addresses the full two-level nested structure, includes items as a source of measurement error, and examines multiple objects of measurement within a single dataset, while also illustrating the computational steps in sufficient detail for replication.

## Method

### Data

This study utilized an empirical data set obtained from a pretest and a posttest administered to persons nested within groups, which were in turn nested within sites. The following notational conventions are used: p for persons (i.e., students), g for groups, s for sites, and i for items. The same instrument was used for both administrations and consisted of 25 Likert-type items ( $n_{i}=25$ ), each with four response categories. The total sample included 1,517 persons ( $n_{p}=1,517$ ). The number of persons within groups ranged from 2 to 62. There were 89 groups ( $n_{g}=89$ ) across 32 sites ( $n_{s}=32$ ). The number of groups per site ranged from 1 to 8.

### Designs

The reporting starts with the $\left(p^{•}:g^{•}:s^{•}\right)\;x\;i^{•}$ design, which represents the full multilevel structure of the data. Additional *G* study designs examined were $\left(p^{•}:s^{•}\right)\;x\;i^{•}$ , $s^{•}\;x\;i^{•}$ , $\left(p^{•}:g^{•}\right)\;x\;i^{•}$ , $g^{•}\;x\;i^{•}$ , and $p^{•}\;x\;i^{•}$ . The site was an aggregate unit of analysis with two levels of nesting, whereas the group represented an aggregate unit of analysis with one level of nesting. The design notation follows Brennan (2001a), in which circles as superscripts indicate the presence of a multivariate variable. In this study, all analyses were conducted using the multivariate *G* theory approach, and this notation is used consistently throughout the article to denote multivariate designs.

Although these designs differ, their analysis follows the same sequential steps. First, the *G* study variance–covariance components are estimated. Then, the *D* study variance–covariance components are computed using the *G* study variance components and sample sizes. Next, the error variances and universe score variance are computed. Finally, the generalizability and dependability coefficients are calculated from the universe score variance and error variance. A key strength of multivariate G theory is that it provides reliability coefficients for composite scores, as well as error covariances and error correlations among levels of the multivariate variable.

Multivariate G theory treats difference scores as composite scores. In this study, the typical definition of difference scores is used to define composite scores such that

(1) $$x_{\mathrm{diff}}=x_{\mathrm{post}}-x_{\mathrm{pre}}\;$$

where $x_{\mathrm{pre}}$ and $x_{\mathrm{post}}$ denote pretest and posttest scores. Then the fixed multivariate variable ( ν) had two levels: pretest and posttest. Thus, the weights (*w*) for pretest and posttest were −1 and +1 , respectively. Given these weights, the variance of the composite score is

(2) $$\sigma_{\mathrm{diff}}^{2}\left(x\right)=\sigma_{\mathrm{pre}}^{2}\left(x\right)+\sigma_{\mathrm{post}}^{2}\left(x\right)-2\sigma_{\mathrm{pre},\mathrm{post}}\left(x\right).$$

In this study, pretest and posttest scores were available for all persons across all groups and sites, resulting in full and symmetric variance–covariance matrices. This is indicated by the use of filled circles as superscripts. That is, a filled circle on a facet denotes that the facet is crossed with the levels of the fixed multivariate variable. Accordingly, all facets in the multivariate designs have filled circles.

The variance–covariance matrices generated for each score effect were utilized to compute variance–covariance matrices for the universe score (i.e., τ ), relative error (i.e., δ ), and absolute error (i.e., Δ ). Once the variance and covariance components are estimated, composite universe score variance ( $\sigma_{\mathrm{diff}}^{2}\left(\tau \right)$ ), composite relative error variance ( $\sigma_{\mathrm{diff}}^{2}\left(\delta \right))$ , and composite absolute error variance ( $\sigma_{\mathrm{diff}}^{2}\left(\Delta \right))$ were computed according to Equation 2. Then the generalizability and dependability coefficients, as well as S/N ratios for composite scores, were computed using the composite universe score and error variances.

For all designs except $\left(p^{•}:g^{•}:s^{•}\right)\;x\;i^{•}$ , mGENOVA (Brennan, 2001b) produced variance–covariance matrices, composite score variance, and coefficients. Since mGENOVA does not support the analysis for $\left(p^{•}:g^{•}:s^{•}\right)\;x\;i^{•}$ , urGENOVA (Brennan, 2001c) and hand calculations in R (R Core Team, 2026) were used for that analysis. These computations and results are presented in the corresponding section and in the supplementary material, including the R scripts and outputs. That section also serves as an illustration of the analysis by showing how generalizability and dependability coefficients for a difference score, which is treated as a composite score, can be computed. For the other designs, analysis steps are not demonstrated, and only results are reported in table form. Although the definition of universe score, relative error, and absolute error varied across the designs, the same general sequence of steps can be followed using the variance–covariance components specific to each design, which is given in Table 1.

#### $\left(p^{•}:g^{•}:s^{•}\right)\;x\;i^{•}\;\mathrm{and}\;\left(P^{•}:G^{•}:s^{•}\right)\;x\;I^{•}$

**Table 1.** Contributing Components in Computation of $\sigma^{2}\left(\tau \right)$ , $\sigma^{2}\left(\delta \right)$ , $\sigma^{2}\left(\Delta \right)$ for Each Design.

Designs	$\sigma^{2}\left(\tau \right)$	$\sigma^{2}\left(\delta \right)$	$\sigma^{2}\left(\Delta \right)$
$\left(p^{•}:g^{•}:s^{•}\right)\;x\;i^{•}$	s	p:g:s,pi:g:s,g:s,gi:s,si	p:g:s,pi:g:s,g:s,gi:s,si,i
$\left(p^{•}:s^{•}\right)\;x\;i^{•}$	s	p:s,pi:s,si	p:s,pi:s,si,i
$s^{•}x\;i^{•}$	s	si	si,i
$\left(p^{•}:g^{•}\right)\;x\;i^{•}$	g	p:g,pi:g,gi	p:g,pi:g,gi,i
$g^{•}\;x\;i^{•}$	g	gi	gi,i
$p^{•}\;x\;i^{•}$	p	pi	pi,i

#### Analysis

**Figure 1.**
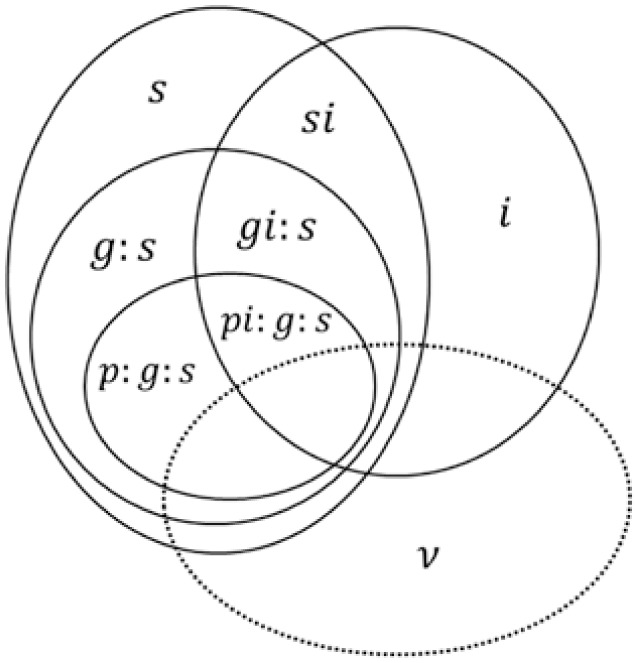
Venn diagram of the $\left(p^{•}:g^{•}:s^{•}\right)\;x\;i^{•}$ design.

Difference scores at the site level, where persons are nested within groups and groups are nested within sites, are modeled using the $\left(p^{•}:g^{•}:s^{•}\right)\;x\;i^{•}$ design, as illustrated in Figure 1. urGENOVA (Brennan, 2001c) can only compute *G* study variance components for the univariate (p:g:s)xi design. The purpose of this study requires covariance estimates between pretest and posttest scores for $\left(p^{•}:g^{•}:s^{•}\right)\;x\;i^{•}$ . To address this limitation, covariance estimates were obtained by combining urGENOVA outputs with the *variance-of-a-sum procedure* (Brennan, 2001a). Specifically, variance components $\sigma^{2}\left(\alpha \right)$ were first estimated using urGENOVA for the posttest scores ( $x_{\mathrm{post}}$ ), pretest scores ( $x_{\mathrm{pre}}$ ), and sum scores ( $x_{\mathrm{sum}}=x_{\mathrm{post}}+x_{\mathrm{pre}}$ ). Then, the covariance component $\sigma_{\mathrm{pre},\mathrm{post}}\left(\alpha \right)$ for each effect was derived using the following equation:

(3) $$\sigma_{\mathrm{pre},\mathrm{post}}\left(\alpha \right)=\frac{\sigma_{\mathrm{sum}}^{2}\left(\alpha \right)-\sigma_{\mathrm{pre}}^{2}\left(\alpha \right)-\sigma_{\mathrm{post}}^{2}\left(\alpha \right)}{2}.$$

Disattenuated correlations between pretest and posttest for each effect were computed according to the following equation:

(4) $$\rho_{\mathrm{pre},\mathrm{post}}\left(\alpha \right)=\;\frac{\sigma_{\mathrm{pre},\mathrm{post}}\left(\alpha \right)}{\sqrt{\sigma_{\mathrm{pre}}^{2}\left(\alpha \right)\;\sigma_{\mathrm{post}}^{2}\left(\alpha \right)}}.$$

Because urGENOVA (Brennan, 2001c) provides variance components but not covariance or correlation components, the covariance and correlation values were derived from these calculations and combined with the variance components to obtain the *G* study variance–covariance matrices. As a result, the following *G* study variance–covariance matrices were obtained:

(5) $${\hat{\Sigma }}_{s}=\left[\begin{array}{c} {\hat{\sigma }}_{\mathrm{pre}}^{2}\left(s\right){\hat{\rho }}_{\mathrm{pre},\;\mathrm{post}}(s)\\ {\hat{\sigma }}_{\mathrm{pre},\;\mathrm{post}}(s){\hat{\sigma }}_{\mathrm{post}}^{2}\left(s\right) \end{array}\right]=\left[\begin{array}{c} 0.01287\;0.43165\\ 0.00763\;0.02431 \end{array}\right],$$

(6) $${\hat{\Sigma }}_{g:s}=\left[\begin{array}{c} {\hat{\sigma }}_{\mathrm{pre}}^{2}\left(g:s\right){\hat{\rho }}_{\mathrm{pre},\;\mathrm{post}}(g:s)\\ {\hat{\sigma }}_{\mathrm{pre},\;\mathrm{post}}(g:s){\hat{\sigma }}_{\mathrm{post}}^{2}\left(g:s\right) \end{array}\right]=\left[\begin{array}{c} 0.02487\;0.68709\\ 0.02130\;0.03866 \end{array}\right],$$

(7) $${\hat{\Sigma }}_{p:g:s}=\left[\begin{array}{c} {\hat{\sigma }}_{\mathrm{pre}}^{2}\left(p:g:s\right){\hat{\rho }}_{\mathrm{pre},\;\mathrm{post}}(p:g:s)\\ {\hat{\sigma }}_{\mathrm{pre},\;\mathrm{post}}(p:g:s){\hat{\sigma }}_{\mathrm{post}}^{2}\left(p:g:s\right) \end{array}\right]=\left[\begin{array}{c} 0.2343\;0.58911\\ 0.14684\;0.26517 \end{array}\right],$$

(8) $${\hat{\Sigma }}_{i}=\left[\begin{array}{c} {\hat{\sigma }}_{\mathrm{pre}}^{2}\left(i\right){\hat{\rho }}_{\mathrm{pre},\;\mathrm{post}}(i)\\ {\hat{\sigma }}_{\mathrm{pre},\;\mathrm{post}}(i){\hat{\sigma }}_{\mathrm{post}}^{2}\left(i\right) \end{array}\right]=\left[\begin{array}{c} 0.12978\;0.98278\\ 0.10158\;0.08231 \end{array}\right],$$

(9) $${\hat{\Sigma }}_{\mathrm{si}}=\left[\begin{array}{c} {\hat{\sigma }}_{\mathrm{pre}}^{2}\left(\mathrm{si}\right){\hat{\rho }}_{\mathrm{pre},\;\mathrm{post}}(\mathrm{si})\\ {\hat{\sigma }}_{\mathrm{pre},\;\mathrm{post}}(\mathrm{si}){\hat{\sigma }}_{\mathrm{post}}^{2}\left(\mathrm{si}\right) \end{array}\right]=\left[\begin{array}{c} 0.01083\;0.76612\\ 0.01096\;0.01888 \end{array}\right],$$

(10) $${\hat{\Sigma }}_{\mathrm{gi}:s}=\left[\begin{array}{c} {\hat{\sigma }}_{\mathrm{pre}}^{2}\left(\mathrm{gi}:s\right){\hat{\rho }}_{\mathrm{pre},\;\mathrm{post}}(\mathrm{gi}:s)\\ {\hat{\sigma }}_{\mathrm{pre},\;\mathrm{post}}(\mathrm{gi}:s){\hat{\sigma }}_{\mathrm{post}}^{2}\left(\mathrm{gi}:s\right) \end{array}\right]=\left[\begin{array}{c} 0.02310\;0.47906\\ 0.00923\;0.01607 \end{array}\right],$$

(11) $${\hat{\Sigma }}_{\mathrm{pi}:g:s}=\left[\begin{array}{c} {\hat{\sigma }}_{\mathrm{pre}}^{2}\left(\mathrm{pi}:g:s\right){\hat{\rho }}_{\mathrm{pre},\;\mathrm{post}}(\mathrm{pi}:g:s)\\ {\hat{\sigma }}_{\mathrm{pre},\;\mathrm{post}}(\mathrm{pi}:g:s){\hat{\sigma }}_{\mathrm{post}}^{2}\left(\mathrm{pi}:g:s\right) \end{array}\right]=\left[\begin{array}{c} 0.57662\;0.25030\\ 0.13315\;0.49081 \end{array}\right].$$

After the *G* study covariance and correlation were calculated for each effect, the random effects *D* study $\left(P^{•}:G^{•}:s^{•}\right)\;x\;I^{•}$ variance–covariance components were computed. $\Sigma_{s}$ is identical, and the rest of the matrices were obtained according to the following equations:

(12) $$\Sigma_{G:s}=\frac{1}{n_{g:s}}\Sigma_{g:s}=\;\left[\begin{array}{c} 0.01059\;0.68709\\ 0.00907\;0.01646\; \end{array}\right],$$

(13) $$\Sigma_{P:G:s}=\frac{1}{n_{p:g:s}}\Sigma_{p:g:s}=\left[\begin{array}{c} 0.02831\;0.58911\\ 0.01774\;0.03204\; \end{array}\right],$$

(14) $$\Sigma_{I}=\frac{1}{n_{i}}\Sigma_{i}=\left[\begin{array}{c} 0.00519\;0.98278\\ 0.00406\;0.00329 \end{array}\right],$$

(15) $$\Sigma_{\mathrm{sI}}=\frac{1}{n_{i}}\Sigma_{\mathrm{si}}=\left[\begin{array}{c} 0.00043\;0.76612\\ 0.00044\;0.00076 \end{array}\right],$$

(16) $$\Sigma_{\mathrm{GI}:s}=\frac{1}{n_{g:s}n_{i}}\Sigma_{\mathrm{gi}:s}=\left[\begin{array}{c} 0.00039\;0.47906\\ 0.00016\;0.00027 \end{array}\right],$$

(17) $$\Sigma_{\mathrm{PI}:G:s}=\frac{1}{n_{p:g:s}n_{g:s}n_{i}}\Sigma_{\mathrm{pi}:g:s}=\left[\begin{array}{c} 0.00119\;0.25030\\ 0.00027\;0.00101 \end{array}\right].$$

In these equations, the *G* study sample sizes were used as divisors in calculating the *D* study variance components. The number of persons within each group and site ( $n_{p:g:s}$ ) was different across groups and sites, ranging from 2 to 62. Similarly, the number of groups within each site ( $n_{g:s}$ ) ranged from 1 to 8. Brennan (2001a) describes this kind of data as unbalanced due to nesting and computes harmonic means to handle it. The same approach was adopted, and harmonic means were used as divisors in computing the *D* study variance and covariance components. The harmonic mean of $n_{p:g:s}$ was calculated in two steps. First, for each site, the harmonic mean of the number of persons across groups was computed, yielding the average number of persons per group for that site. Then, the harmonic mean of these site-level harmonic means was computed. This process resulted in 8.277. To determine the average number of groups per site, the harmonic mean of the number of groups across all sites was computed. This process resulted in 2.349. The number of items ( $n_{i})$ was the same across all facets, which was 25.

The next step was to compute the variance–covariance components of the $\Sigma_{\tau },\;\Sigma_{\delta }$ , and $\Sigma_{\Delta }$ and generalizability and dependability coefficients. For the random effects $\left(P^{•}:G^{•}:s^{•}\right)\;x\;I^{•}$ design, sites are the object of measurement, and it implies $\Sigma_{\tau }=\Sigma_{s}$ such that

(18) $${\hat{\Sigma }}_{\tau }=\left[\begin{array}{c} {\hat{\sigma }}_{\mathrm{pre}}^{2}\left(\tau \right){\hat{\rho }}_{\mathrm{pre},\;\mathrm{post}}(\tau )\\ {\hat{\sigma }}_{\mathrm{pre},\;\mathrm{post}}(\tau ){\hat{\sigma }}_{\mathrm{post}}^{2}\left(\tau \right) \end{array}\right]=\left[\begin{array}{c} {\hat{\sigma }}_{\mathrm{pre}}^{2}\left(s\right){\hat{\rho }}_{\mathrm{pre},\;\mathrm{post}}(s)\\ {\hat{\sigma }}_{\mathrm{pre},\;\mathrm{post}}(s){\hat{\sigma }}_{\mathrm{post}}^{2}\left(s\right) \end{array}\right]=\left[\begin{array}{c} 0.01287\;0.43192\\ 0.00764\;0.02431 \end{array}\right].$$

$\Sigma_{\delta }$ and $\Sigma_{\Delta }$ variance–covariance matrices were defined as

(19) $$\Sigma_{\delta }=\Sigma_{P:G:s}+\Sigma_{\mathrm{PI}:G:s}+\Sigma_{G:s}+\Sigma_{\mathrm{GI}:s}+\Sigma_{\mathrm{sI}},$$

(20) $$\Sigma_{\Delta }=\Sigma_{P:G:s}+\Sigma_{\mathrm{PI}:G:s}+\Sigma_{G:s}+\Sigma_{\mathrm{GI}:s}+\Sigma_{\mathrm{sI}}+\Sigma_{I}.$$

Applying the D study variance–covariance components reported in matrices in Equations 12–17 into Equations 19 and 20 resulted in a relative and absolute error variance–covariance matrix,

(21) $${\hat{\Sigma }}_{\delta }=\left[\begin{array}{c} {\hat{\sigma }}_{\mathrm{pre}}^{2}\left(\delta \right){\hat{\rho }}_{\mathrm{pre},\;\mathrm{post}}(\delta )\\ {\hat{\sigma }}_{\mathrm{pre},\;\mathrm{post}}(\delta ){\hat{\sigma }}_{\mathrm{post}}^{2}\left(\delta \right) \end{array}\right]=\left[\begin{array}{c} 0.04091\;0.60875\\ 0.02768\;0.05054 \end{array}\right],$$

(22) $${\hat{\Sigma }}_{\Delta }=\left[\begin{array}{c} {\hat{\sigma }}_{\mathrm{pre}}^{2}\left(\Delta \right){\hat{\rho }}_{\mathrm{pre},\;\mathrm{post}}(\Delta )\\ {\hat{\sigma }}_{\mathrm{pre},\;\mathrm{post}}(\Delta ){\hat{\sigma }}_{\mathrm{post}}^{2}\left(\Delta \right) \end{array}\right]=\left[\begin{array}{c} 0.04610\;0.63720\\ 0.03174\;0.05383 \end{array}\right].$$

Universe score variance and relative and absolute error variance are used to compute the generalizability and dependability coefficients, which are defined as:

(23) $$E\rho^{2}=\frac{\sigma^{2}\left(\tau \right)}{\sigma^{2}\left(\tau \right)+\sigma^{2}\left(\delta \right)},$$

(24) $$\Phi =\frac{\sigma^{2}\left(\tau \right)}{\sigma^{2}\left(\tau \right)+\sigma^{2}\left(\Delta \right)}.$$

The S/N represents the ratio of universe score variance to error variance, such that

(25) $$S/N(\delta )=\frac{\sigma^{2}\left(\tau \right)}{\sigma^{2}\left(\delta \right)},$$

(26) $$S/N(\Delta )=\frac{\sigma^{2}\left(\tau \right)}{\sigma^{2}\left(\Delta \right)}.$$

Substituting the ${\hat{\sigma }}_{\mathrm{pre}}^{2}\left(\tau \right),$ ${\hat{\sigma }}_{\mathrm{pre}}^{2}\left(\delta \right)$ , and ${\hat{\sigma }}_{\mathrm{pre}}^{2}\left(\Delta \right)$ into Equations 23–26 yielded the following values for pretest scores, such that

(27) $$E{\hat{\rho }}_{\mathrm{pre}}^{2}=\frac{{\hat{\sigma }}_{\mathrm{pre}}^{2}\left(\tau \right)}{{\hat{\sigma }}_{\mathrm{pre}}^{2}\left(\tau \right)+{\hat{\sigma }}_{\mathrm{pre}}^{2}\left(\delta \right)}=\frac{0.01287}{0.01287+0.04091}=0.23931,$$

(28) $${\hat{\Phi }}_{\mathrm{pre}}=\frac{{\hat{\sigma }}_{\mathrm{pre}}^{2}\left(\tau \right)}{{\hat{\sigma }}_{\mathrm{pre}}^{2}\left(\tau \right)+{\hat{\sigma }}_{\mathrm{pre}}^{2}\left(\Delta \right)}=\;\frac{0.01287}{0.01287+0.04610\;}=\;0.21825,$$

(29) $$S/N{\left(\delta \right)}_{\mathrm{pre}}=\frac{{\hat{\sigma }}_{\mathrm{pre}}^{2}\left(\tau \right)}{{\hat{\sigma }}_{\mathrm{pre}}^{2}\left(\delta \right)}=\;\frac{0.01287}{0.04091\;}=0.31459,$$

(30) $$S/N{\left(\Delta \right)}_{\mathrm{pre}}=\frac{{\hat{\sigma }}_{\mathrm{pre}}^{2}\left(\tau \right)}{{\hat{\sigma }}_{\mathrm{pre}}^{2}\left(\Delta \right)}=\;\frac{0.01287}{0.04610\;}=0.27918.$$

These coefficients can be calculated for posttest scores in a similar way, such that $E{\hat{\rho }}_{\mathrm{post}}^{2}=0.32478,$ ${\hat{\Phi }}_{\mathrm{post}}=0.31111$ , $S/N{\left(\delta \right)}_{\mathrm{post}}=\;0.48101,$ and $S/N{\left(\Delta \right)}_{\mathrm{post}}=0.45161$ . Up to this point, variance–covariance matrices and coefficients for individual variables have been produced. Composite universe score variance, error variances, and coefficients were computed by substituting the variance–covariance components of the matrices given in Equations 18, 21, and 22 in Equation 2. Specifically, the composite universe score variance was computed as:

(31) $$\begin{array}{c} {\hat{\sigma }}_{\mathrm{diff}}^{2}\left(\tau \right)={\hat{\sigma }}_{\mathrm{pre}}^{2}\left(\tau \right)+{\hat{\sigma }}_{\mathrm{post}}^{2}\left(\tau \right)-2{\hat{\sigma }}_{\mathrm{pre},\;\mathrm{post}}(\tau )\\ =0.01287+0.02431-2*0.00764=0.0219\;. \end{array}$$

Similarly, the composite relative and absolute error variances were computed as:

(32) $$\begin{array}{c} {\hat{\sigma }}_{\mathrm{diff}}^{2}\left(\delta \right)={\hat{\sigma }}_{\mathrm{pre}}^{2}\left(\delta \right)+{\hat{\sigma }}_{\mathrm{post}}^{2}\left(\delta \right)-2{\hat{\sigma }}_{\mathrm{pre},\;\mathrm{post}}(\delta )\\ \;=0.04091+0.05054-2*0.02768=0.03609, \end{array}$$

(33) $$\begin{array}{c} {\hat{\sigma }}_{\mathrm{diff}}^{2}\left(\Delta \right)={\hat{\sigma }}_{\mathrm{pre}}^{2}\left(\Delta \right)+{\hat{\sigma }}_{\mathrm{post}}^{2}\left(\Delta \right)-2{\hat{\sigma }}_{\mathrm{pre},\;\mathrm{post}}(\Delta )\\ =\;0.04610+0.05383-2*0.03174=0.03645. \end{array}$$

The composite score coefficients were then calculated using ${\hat{\sigma }}_{\mathrm{diff}}^{2}\left(\tau \right),$ ${\hat{\sigma }}_{\mathrm{diff}}^{2}\left(\delta \right)$ , and ${\hat{\sigma }}_{\mathrm{diff}}^{2}\left(\Delta \right)$ :

(34) $$E{\hat{\rho }}_{\mathrm{diff}}^{2}=\frac{{\hat{\sigma }}_{\mathrm{diff}}^{2}\left(\tau \right)}{{\hat{\sigma }}_{\mathrm{diff}}^{2}\left(\tau \right)+{\hat{\sigma }}_{\mathrm{diff}}^{2}\left(\delta \right)}=\;\frac{0.0219}{0.0219+0.03609\;}=\;0.37765,$$

(35) $${\hat{\Phi }}_{\mathrm{diff}}=\frac{{\hat{\sigma }}_{\mathrm{diff}}^{2}\left(\tau \right)}{{\hat{\sigma }}_{\mathrm{diff}}^{2}\left(\tau \right)+{\hat{\sigma }}_{\mathrm{diff}}^{2}\left(\Delta \right)}=\frac{0.0219}{0.0219+0.03645}=0.37532.$$

(36) $$S/N{\left(\delta \right)}_{\mathrm{diff}}=\frac{{\hat{\sigma }}_{\mathrm{diff}}^{2}\left(\tau \right)}{{\hat{\sigma }}_{\mathrm{diff}}^{2}\left(\delta \right)}=\;\frac{0.0219}{0.03609\;}=0.60682,$$

(37) $$S/N{\left(\Delta \right)}_{\mathrm{diff}}=\frac{{\hat{\sigma }}_{\mathrm{diff}}^{2}\left(\tau \right)}{{\hat{\sigma }}_{\mathrm{diff}}^{2}\left(\Delta \right)}=\;\frac{0.0219}{0.03645\;}=0.60082.$$

##### Results

The variance–covariance components of $\left(p^{•}:g^{•}:s^{•}\right)\;x\;i^{•}$ are presented in matrices in Equations 5–11. The estimated variance component of posttest scores for sites ( ${\hat{\sigma }}_{\mathrm{post}}^{2}(s)$ ) was higher than that of pretest scores ( ${\hat{\sigma }}_{\mathrm{pre}}^{2}(s)$ ). Similarly, the posttest variance components for g:s , p:g:s , and si were larger than their corresponding pretest variance components. In contrast, the posttest variance components for i , gi:s , and pi:g:s were smaller than components for pretest scores. Despite these differences in magnitude, the pattern of variance components was generally consistent across pretest and posttest scores. For instance, the variance component for pi:g:s was the largest estimated variance component. This was expected, as it captures the residual variance, encompassing all sources of variation that is not represented in the universe of admissible observations (Brennan, 2001a). The second largest variance component was for p:g:s , indicating substantial variability among persons nested within groups and sites. This result was expected given the presence of two nesting facets, which would otherwise have contributed to crossed sources of variation.

The estimated disattenuated correlation between pretest and posttest scores for items (i.e., ${\hat{\rho }}_{\mathrm{pre},\mathrm{post}}(i)$ ) was very high, as presented in Equation 8, suggesting a strong linear relationship between item scores across the two occasions. The correlation for the site-by-item interaction ( ${\hat{\rho }}_{\mathrm{pre},\mathrm{post}}(\mathrm{si})$ ) was relatively high, suggesting that the rank ordering of sites by item remained largely consistent from pretest to posttest. By contrast, ${\hat{\rho }}_{\mathrm{pre},\mathrm{post}}(s)$ was the lowest among all components except for the residual term pi:g:s . This suggests that the rank ordering of sites’ pretest mean scores and posttest mean scores was not strongly preserved. Notably, ${\hat{\sigma }}_{\mathrm{post}}^{2}(s)$ was approximately twice as large as ${\hat{\sigma }}_{\mathrm{pre}}^{2}(s)$ . These findings indicate that the intervention contributed to an increase in the variability of scores at the site level. In addition, ${\hat{\sigma }}_{\mathrm{pre}\;}^{2}(p:g:s)$ was almost 10 times greater than ${\hat{\sigma }}_{\mathrm{pre}\;}^{2}(g:s)$ , and ${\hat{\sigma }}_{\mathrm{pre}\;}^{2}(g:s)$ was almost twice as large as ${\hat{\sigma }}_{\mathrm{pre}\;}^{2}(s)$ . Moreover, the difference between pretest and posttest variance components was larger for s than for g:s . Similarly, the difference was larger for g:s than for p:g:s . This suggests that the intervention had a more substantial effect on aggregated levels of measurement in terms of variance components.

The variance and covariance components of the *D* study random effects design, $\left(P^{•}:G^{•}:s^{•}\right)\;x\;I^{•}$ , are reported in Equations 12–17. In this design, sites are the object of measurement, and the universe of generalization involves persons, groups, and items. This means that generalization was made from site scores obtained from specific items answered by specific persons within specific groups to the site scores that would be obtained from randomly sampled items, persons, and groups from infinitely large universes. In both the universe of generalization and the *D* study design, items were crossed with sites, while groups were nested within sites, and persons were nested within groups. This can be interpreted as each site’s observed mean difference score was associated with approximately two groups per site, with approximately eight persons within a group since *D* study divisors are 2.349 and 8.277.

Estimates of the variance–covariance components of the $\Sigma_{\tau },\Sigma_{\delta }$ , and $\Sigma_{\Delta }$ and coefficients are reported in Equations 18, 21, and 22. The estimated correlation between site mean universe scores ( ${\hat{\rho }}_{\mathrm{pre},\mathrm{post}}\left(\tau \right)$ ) was positive but relatively low. This suggests that the rank ordering of sites differed between pretest and posttest. This pattern is commonly observed with difference scores and is one of the key reasons they often exhibit low reliability. Both relative and absolute error correlations were nonzero and similar in magnitude. This was expected, as the variance of items was very small for both administrations. The largest covariance component contributing to both ${\hat{\rho }}_{\mathrm{pre},\mathrm{post}}\left(\delta \right)$ and ${\hat{\rho }}_{\mathrm{pre},\mathrm{post}}\left(\Delta \right)$ was P:G:s , and the second largest one was G:s .

As presented in Equation 34, $E{\hat{\rho }}_{\mathrm{diff}}^{2}$ suggests that the rank ordering of sites based on mean difference scores is likely to vary considerably over randomly sampled items, persons, and groups from infinitely large universes. Similarly, the small value of ${\hat{\Phi }}_{\mathrm{diff}}$ , given in Equation 35, suggests that for a randomly selected site, the absolute magnitude of the observed difference score is not a dependable estimate of the true difference score. S/N is the ratio of universe score variance to error variance and can range from 0 to infinity. Both S/N(δ) and S/N(Δ) , presented in Equations 36 and 37, indicate that the universe score variance was half the error variance. This suggests that, under the current measurement conditions, it is quite difficult to detect meaningful differences among sites.

### $\left(p^{•}:s^{•}\right)\;x\;i^{•}$ **and** $\left(P^{•}:s^{•}\right)\;x\;I^{•}$

**Figure 2.**
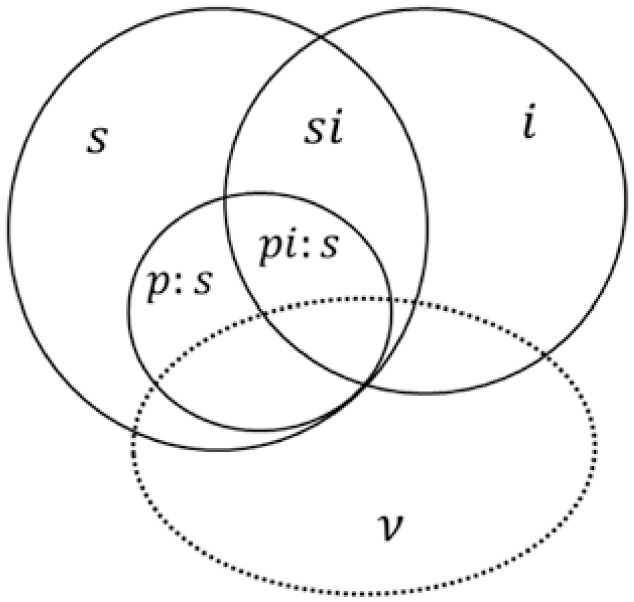
Venn diagram of $\left(p^{•}:s^{•}\right)\;x\;i^{•}$ .

When the group facet was omitted, the *G* study design simplified to $\left(p^{•}:s^{•}\right)\;x\;i^{•}$ . Figure 2 provides a Venn diagram representation of this design. The number of persons within sites ( $n_{p:s}$ ) ranged from 2 to 139, with a harmonic mean of 21.7. The number of sites ( $n_{s})$ and items ( $n_{i})$ were 32 and 25, respectively. Estimates of the *G* study variance and covariance components for both pretest and posttest scores are reported in Table 2.

**Table 2.** Variance and Covariance Components for $\left(p^{•}:s^{•}\right)\;x\;i^{•}$ and $\left(P^{•}:s^{•}\right)\;x\;I^{•}$ .

$\left(p^{•}:s^{•}\right)\;x\;i^{•}$					$\left(P^{•}:s^{•}\right)\;x\;I^{•}$				
α	${\hat{\sigma }}_{\mathrm{pre}}^{2}$	${\hat{\sigma }}_{\mathrm{post}}^{2}$	${\hat{\sigma }}_{\mathrm{pre},\mathrm{post}}$	${\hat{\rho }}_{\mathrm{pre},\mathrm{pos}}$	n	α	${\hat{\sigma }}_{\mathrm{pre}}^{2}$	${\hat{\sigma }}_{\mathrm{post}}^{2}$	${\hat{\sigma }}_{\mathrm{pre},\mathrm{post}}$
*s*	0.024	0.042	0.017	0.543		*s*	0.024	0.042	0.017
*p:s*	0.248	0.287	0.159	0.595	21.73	*P:s*	0.011	0.013	0.007
*i*	0.130	0.082	0.102	0.983	25.0	*I*	0.005	0.003	0.004
*si*	0.021	0.026	0.015	0.642	25.0	*sI*	0.001	0.001	0.001
*pi:s*	0.589	0.500	0.138	0.255	543.36	*PI:s*	0.001	0.001	0.000

When the group facet is ignored, the within-group variability contributes to the variance of other facets. This contribution can be directly observed through the increase in the variance of s , si , and the residual term compared to the ones obtained from $\left(p^{•}:g^{•}:s^{•}\right)\;x\;i^{•}$ . On the contrary, ${\hat{\sigma }}^{2}\left(i\right)$ values remained the same since g was a nested facet within s . Moreover, both designs share certain commonalities. The variance of the facet nested within the object of measurement was the largest. For both pretest and posttest scores, the largest variance component, aside from the residual, was for p:s . ${\hat{\sigma }}_{\mathrm{pre}\;}^{2}(p:s)$ was almost 10 times larger than ${\hat{\sigma }}_{\mathrm{pre}\;}^{2}(s)$ , and ${\hat{\sigma }}_{\mathrm{post}\;}^{2}(p:s)$ was almost 7 times larger than ${\hat{\sigma }}_{\mathrm{post}\;}^{2}(s)$ . There was a second nested effect in the $\left(p^{•}:g^{•}:s^{•}\right)\;x\;i^{•}$ , which produced the largest variance for p:g:s of both pretest and posttest. The variance components, except for i and the residual term, were all larger for posttest scores than for pretest scores. For i and the residual, it was the opposite.

The *D* study random effects design, with sites as the object of measurement, is denoted as $\left(P^{•}:s^{•}\right)\;x\;I^{•}$ . The universe of generalization involves persons and items. The estimated *D* study variance and covariance components are summarized in Table 2. The universe score variance–covariance components, ${\hat{\sigma }}_{\mathrm{pre}\;}^{2}(s)=0.024$ and ${\hat{\sigma }}_{\mathrm{post}\;}^{2}(s)=0.042$ , were larger than the ones obtained for $\left(P^{•}:G^{•}:s^{•}\right)\;x\;I^{•}$ . Variance and covariance components for I were identical with the $\left(P^{•}:G^{•}:s^{•}\right)\;x\;I^{•}$ while there was no consistent increase for the other facets since divisors were also changed.

The *D* study variance and covariance components given in Table 2 were used to compute the error variances and coefficients, which are reported in Table 3. When the group facet is ignored, the within-group variability contributes to the ${\hat{\sigma }}^{2}\left(s\right)$ , and thus the universe score variance ${\hat{\sigma }}^{2}\left(\tau \right)$ . Moreover, ${\hat{\sigma }}^{2}\left(\delta \right)$ and ${\hat{\sigma }}^{2}\left(\Delta \right)$ decreased in magnitude, which was expected since the contribution of group variability within sites could not be included in the estimation of error variances. This resulted in increases in $\hat{\Phi }$ , $E{\hat{\rho }}^{2}$ , S/N(δ) , and S/N(Δ) . The universe score correlation ${\hat{\rho }}_{\mathrm{pre},\mathrm{post}}\left(\tau \right)=0.543$ , as presented in Table 3, for this design was notably larger than that for the previous $\left(P^{•}:G^{•}:s^{•}\right)\;x\;I^{•}$ design. This shows that ignoring the group facet resulted in inflated correlation estimates of universe scores.

**Table 3.** *D* Study Results for $\left(P^{•}:s^{•}\right)\;x\;I^{•}$ for $x_{\mathrm{pre}}$ , $x_{\mathrm{post}}$ , and the Composite Score $x_{\mathrm{diff}}$

Statistic	$x_{\mathrm{pre}}$	$x_{\mathrm{post}}$	$x_{\mathrm{diff}}$	${\hat{\rho }}_{\mathrm{pre},\mathrm{post}}$
${\hat{\sigma }}^{2}\left(\tau \right)$	0.024	0.042	0.031	0.543
${\hat{\sigma }}^{2}\left(\delta \right)$	0.013	0.015	0.012	0.574
${\hat{\sigma }}^{2}\left(\Delta \right)$	0.019	0.018	0.013	0.661
$E{\hat{\rho }}^{2}$	0.643	0.733	0.720	
$\hat{\Phi }$	0.564	0.693	0.714	
S/N(δ)	1.800	2.749	2.569	
S/N(Δ)	1.296	2.258	2.495	

Excluding the g facet from the design nearly doubled the reliability coefficients. $E{\hat{\rho }}_{\mathrm{diff}}^{2}$ was relatively high suggesting that the rank ordering of sites in terms of mean difference scores was consistent. Similarly, the relatively large value of ${\hat{\Phi }}_{\mathrm{diff}}$ suggests that, for a randomly selected site, the absolute magnitude of the observed difference score is a dependable estimate of the true difference score. Both S/N(δ) and S/N(Δ) indicate that the universe score variance was 2.5 times larger than the error variance. This suggests that detecting differences among sites is relatively easier with this measurement procedure compared to $\left(P^{•}:G^{•}:s^{•}\right)\;x\;I^{•}$ . However, these coefficients were overestimated, as the data structure was misrepresented in this simplified design. When sites are the object of measurement, both persons and groups should be treated as facets in the universe of generalization. By omitting the group facet, this design failed to properly account for group-level variability, which contributes to the error variance, leading to inflated estimates of reliability coefficients.

### $s^{•}\;x\;i^{•}$ **and** $s^{•}\;x\;I^{•}$

**Figure 3.**
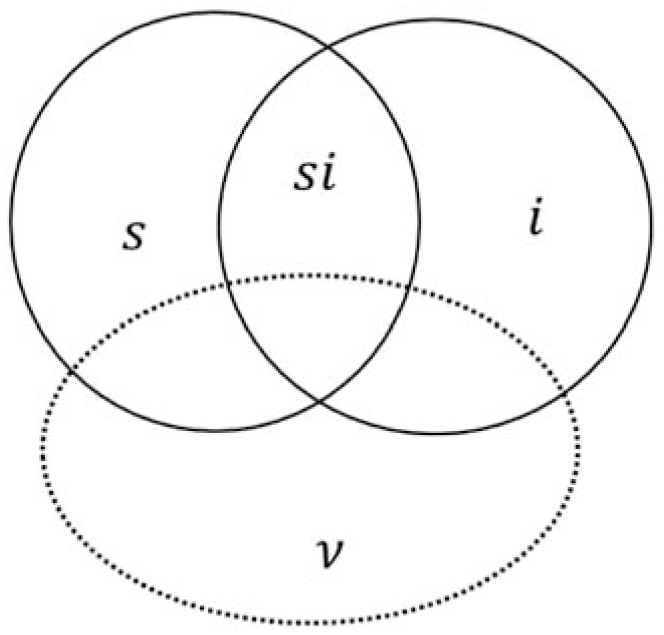
Venn diagram of $s^{•}\;x\;i^{•}$ .

In the *G* study design $s^{•}\;x\;i^{•}$ , both the group and person facets were omitted, and sites served as the object of measurement (see Figure 3). The number of sites ( $n_{s}$ ) was 32, and the number of items ( $n_{i}$ ) was 25. The *G* study variance and covariance components are given in Table 4. When both group and person facets were ignored, only site, item, and residual were left in the model. Variability among groups and persons contributed to the variance of the site facet. This contribution could be directly observed through the increased variance of s and si . For the site facet, ${\hat{\sigma }}_{\mathrm{post}}^{2}>{\hat{\sigma }}_{\mathrm{pre}}^{2}$ , while for the item facet and residual, ${\hat{\sigma }}_{\mathrm{pre}}^{2}>{\hat{\sigma }}_{\mathrm{post}}^{2}$ . This finding was parallel with the fact that the variance components for i and the residual term were larger for pretest scores than for posttest scores, and it was the opposite for the other variance components. In addition, ${\hat{\rho }}_{\mathrm{pre},\mathrm{post}}(i)$ was very large, while ${\hat{\rho }}_{\mathrm{pre},\mathrm{post}}(s)$ was moderate in magnitude.

**Table 4.** Variance and Covariance Components for $s^{•}\;x\;i^{•}$ and $s^{•}\;x\;I^{•}$ .

$s^{•}\;x\;i^{•}$					$s^{•}\;x\;I^{•}$				
α	${\hat{\sigma }}_{\mathrm{pre}}^{2}$	${\hat{\sigma }}_{\mathrm{post}}^{2}$	${\hat{\sigma }}_{\mathrm{pre},\mathrm{post}}$	${\hat{\rho }}_{\mathrm{pre},\mathrm{post}}$	n	α	${\hat{\sigma }}_{\mathrm{pre}}^{2}$	${\hat{\sigma }}_{\mathrm{post}}^{2}$	${\hat{\sigma }}_{\mathrm{pre},\mathrm{post}}$
*s*	0.051	0.079	0.037	0.579		*s*	0.051	0.079	0.037
*i*	0.126	0.077	0.096	0.977	25.0	*I*	0.005	0.003	0.004
*si*	0.073	0.054	0.026	0.412	25.0	*sI*	0.003	0.002	0.001

The variance and covariance components for the *D* study $s^{•}\;x\;I^{•}$ are illustrated in Table 4. The correlation of site universe scores between pretest and posttest was ${\hat{\rho }}_{\mathrm{pre},\mathrm{post}}\left(\tau \right)=0.579$ . Relatively large values for $E{\hat{\rho }}_{\mathrm{diff}}^{2}$ , ${\hat{\Phi }}_{\mathrm{diff}}$ , S/N(δ) , and S/N(Δ) coefficients were obtained, which are shown in Table 5. Again, the person and group facets were not explicitly modeled in this design, which has led to inflated estimates of these coefficients.

**Table 5.** *D* Study Results for $s^{•}\;x\;I^{•}$ for $x_{\mathrm{pre}}$ , $x_{\mathrm{post}}$ , and the Composite Score $x_{\mathrm{diff}}$ .

Statistic	$x_{\mathrm{pre}}$	$x_{\mathrm{post}}$	$x_{\mathrm{diff}}$	${\hat{\rho }}_{\mathrm{pre},\mathrm{post}}$
${\hat{\sigma }}^{2}\left(\tau \right)$	0.051	0.079	0.057	0.579
${\hat{\sigma }}^{2}\left(\delta \right)$	0.003	0.002	0.003	0.412
${\hat{\sigma }}^{2}\left(\Delta \right)$	0.008	0.005	0.003	0.757
$E{\hat{\rho }}^{2}$	0.946	0.974	0.950	
$\hat{\Phi }$	0.866	0.938	0.943	
S/N(δ)	17.564	36.966	18.851	
S/N(Δ)	6.446	15.164	16.543	

### $\left(p^{•}:g^{•}\right)\;x\;i^{•}$ **and** $\left(P^{•}:g^{•}\right)\;x\;I^{•}$

**Figure 4.**
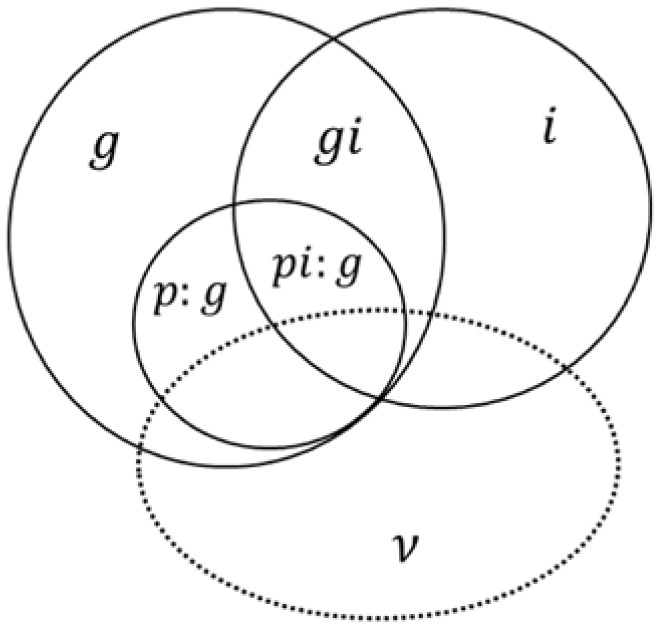
Venn diagram of $\left(p^{•}:g^{•}\right)\;x\;i^{•}$ .

The *G* study design $\left(p^{•}:g^{•}\right)\;x\;i^{•}$ represents persons nested within groups and crossed with items, as illustrated in Figure 4. The number of persons within the groups ( $n_{p:g}$ ) ranged from 2 to 86, with a harmonic mean of 8.119. The number of groups ( $n_{g})$ and items ( $n_{i})$ were 89 and 25, respectively. The estimated *G* study variance and covariance components for both pretest and posttest scores are given in Table 6.

**Table 6.** Variance and Covariance Components for $\left(p^{•}:g^{•}\right)\;x\;i^{•}$ and $\left(P^{•}:g^{•}\right)\;x\;I^{•}$ .

$\left(p^{•}:g^{•}\right)\;x\;i^{•}$					$\left(P^{•}:g^{•}\right)\;x\;I^{•}$				
α	${\hat{\sigma }}_{\mathrm{pre}}^{2}$	${\hat{\sigma }}_{\mathrm{post}}^{2}$	${\hat{\sigma }}_{\mathrm{pre},\mathrm{post}}$	${\hat{\rho }}_{\mathrm{pre},\mathrm{post}}$	n	α	${\hat{\sigma }}_{\mathrm{pre}}^{2}$	${\hat{\sigma }}_{\mathrm{post}}^{2}$	${\hat{\sigma }}_{\mathrm{pre},\mathrm{post}}$
*g*	0.037	0.062	0.029	0.595		*g*	0.037	0.062	0.029
*p:g*	0.234	0.265	0.147	0.589	8.12	*P:g*	0.029	0.033	0.018
*i*	0.130	0.083	0.102	0.982	25.0	*I*	0.005	0.003	0.004
*gi*	0.034	0.034	0.020	0.584	25.0	*gI*	0.001	0.001	0.001
*pi:g*	0.577	0.491	0.133	0.250	202.97	*PI:g*	0.003	0.002	0.001

The patterns among the variance components of this design mirrored the results obtained from the $\left(p^{•}:s^{•}\right)\;x\;i^{•}$ . The variance components for g , p:g , and gi were larger for posttest scores than for pretest scores, while i and pi:g components for posttest scores were smaller than those for pretest scores. For both pretest and posttest scores, the largest variance component other than the residual was for p:g . ${\hat{\sigma }}_{\mathrm{pre}}^{2}(p:g)$ was almost seven times greater than ${\hat{\sigma }}_{\mathrm{pre}\;}^{2}(g)$ , which is the smallest among the pretest variances. ${\hat{\sigma }}_{\mathrm{post}}^{2}\left(g\right)$ was larger than ${\hat{\sigma }}_{\mathrm{pre}}^{2}(g)$ , while ${\hat{\sigma }}_{\mathrm{post}}^{2}\left(i\right)$ was substantially smaller than ${\hat{\sigma }}_{\mathrm{pre}}^{2}\left(i\right)$ . This suggests that group mean scores were more variable in the posttest, whereas item scores were more similar in the posttest.

The *D* study random effects design with groups as the object of measurement was $\left(P^{•}:g^{•}\right)\;x\;I^{•}$ . The estimated D study variance and covariance components are summarized in Table 6. The universe of generalization involved persons and items. In both the universe of generalization and the *D* study design, items were crossed with groups, and persons were nested within groups. The estimated universe score variance for group mean pretest and posttest scores were ${\hat{\sigma }}_{\mathrm{pre}}^{2}\left(\tau \right)=0.037$ and ${\hat{\sigma }}_{\mathrm{post}}^{2}\left(\tau \right)=0.062$ . The correlation between group mean universe scores for pretest and posttest ( ${\hat{\rho }}_{\mathrm{pre},\mathrm{post}}\left(\tau \right)=0.595$ ) was moderate in size and positive.

The *D* study variance and covariance components presented in Table 6 were used to compute the error variances and coefficients, which are reported in Table 7. Consistent with previous designs, the relative and absolute error variances were similar in magnitude, and this resulted in similar values between ${\hat{\Phi }}_{\mathrm{diff}}=0.579$ and $E{\hat{\rho }}_{\mathrm{diff}}^{2}=0.582$ . In addition, both S/N(δ) and S/N(Δ) indicate that the universe score variance was quite low compared to the error variance. This suggests that detecting differences among randomly selected groups is relatively difficult with this measurement procedure.

**Table 7.** *D* Study Results for $\left(P^{•}:g^{•}\right)\;x\;I^{•}$ for $x_{\mathrm{pre}}$ , $x_{\mathrm{post}}$ , and the Composite Score $x_{\mathrm{diff}}$ .

Statistic	$x_{\mathrm{pre}}$	$x_{\mathrm{post}}$	$x_{\mathrm{diff}}$	${\hat{\rho }}_{\mathrm{pre},\mathrm{post}}$
${\hat{\sigma }}^{2}\left(\tau \right)$	0.037	0.062	0.042	0.595
${\hat{\sigma }}^{2}\left(\delta \right)$	0.033	0.036	0.030	0.563
${\hat{\sigma }}^{2}\left(\Delta \right)$	0.038	0.040	0.031	0.605
$E{\hat{\rho }}^{2}$	0.531	0.631	0.582	
$\hat{\Phi }$	0.495	0.611	0.579	
S/N(δ)	1.132	1.711	1.390	
S/N(Δ)	0.978	1.568	1.373	

### $g^{•}\;x\;i^{•}$ **and** $g^{•}\;x\;I^{•}$

**Figure 5.**
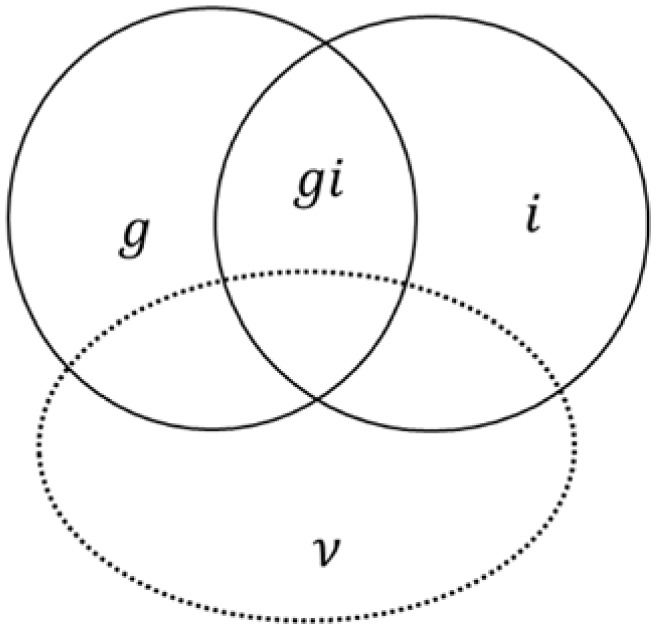
Venn diagram of $g^{•}\;x\;i^{•}$ .

When the person facet was ignored in $\left(p^{•}:g^{•}\right)\;x\;i^{•}$ , the *G* study design becomes $g^{•}\;x\;i^{•}$ , which is presented in Figure 5. The number of groups and the number of items were identical to those in the previous design. Estimates of the *G* study variance and covariance components for both pretest and posttest scores are presented in Table 8. Findings from the $g^{•}\;x\;i^{•}$ paralleled those observed in the $s^{•}\;x\;i^{•}$ design such that the variance components for i and residual term were larger for pretest scores than for posttest scores, and it was the opposite for the g . ${\hat{\rho }}_{\mathrm{pre},\mathrm{post}}(i)$ was very high, and ${\hat{\rho }}_{\mathrm{pre},\mathrm{post}}(g)$ was moderate.

**Table 8.** Variance and Covariance Components for $g^{•}\;x\;i^{•}$ and $g^{•}\;x\;I^{•}$ .

$g^{•}\;x\;i^{•}$					$g^{•}\;x\;I^{•}$				
α	${\hat{\sigma }}_{\mathrm{pre}}^{2}$	${\hat{\sigma }}_{\mathrm{post}}^{2}$	${\hat{\sigma }}_{\mathrm{pre},\mathrm{post}}$	${\hat{\rho }}_{\mathrm{pre},\mathrm{post}}$	n	α	${\hat{\sigma }}_{\mathrm{pre}}^{2}$	${\hat{\sigma }}_{\mathrm{post}}^{2}$	${\hat{\sigma }}_{\mathrm{pre},\mathrm{post}}$
*g*	0.064	0.102	0.041	0.505		*g*	0.064	0.102	0.041
*i*	0.135	0.085	0.105	0.979	25.0	*I*	0.005	0.003	0.004
*gi*	0.129	0.107	0.046	0.389	25.0	*gI*	0.005	0.004	0.002

The *D* study random effects design, with groups as the object of measurement, was $g^{•}\;x\;I^{•}$ . Estimates of the *D* study variance and covariance components are summarized in Table 8. The universe score variances were ${\hat{\sigma }}_{\mathrm{pre}}^{2}\left(\tau \right)=0.064$ and ${\hat{\sigma }}_{\mathrm{post}}^{2}\left(\tau \right)=0.102$ for the groups’ pretest and posttest scores, respectively. The correlation between group universe scores for the pretest and posttest was ${\hat{\rho }}_{\mathrm{prepost}}\left(\tau \right)=0.505$ . Compared to the $\left(P^{•}:g^{•}\right)\;x\;I^{•}$ design, the values of ${\hat{\sigma }}^{2}\left(\delta \right)$ and ${\hat{\sigma }}^{2}\left(\Delta \right)$ decreased, and those of ${\hat{\sigma }}^{2}\left(\tau \right)$ , ${\hat{\Phi }}_{\mathrm{diff}}$ , $E{\hat{\rho }}_{\mathrm{diff}}^{2}$ , S/N(δ) , and S/N(Δ) increased, which can be seen in Table 9. These inflated coefficients are likely attributable to the omission of the person facet in this design.

**Table 9.** *D* Study Results for $g^{•}\;x\;I^{•}$ for $x_{\mathrm{pre}}$ , $x_{\mathrm{post}}$ , and the Composite Score $x_{\mathrm{diff}}$ .

Statistic	$x_{\mathrm{pre}}$	$x_{\mathrm{post}}$	$x_{\mathrm{diff}}$	${\hat{\rho }}_{\mathrm{pre},\mathrm{post}}$
${\hat{\sigma }}^{2}\left(\tau \right)$	0.064	0.102	0.084	0.505
${\hat{\sigma }}^{2}\left(\delta \right)$	0.005	0.004	0.006	0.389
${\hat{\sigma }}^{2}\left(\Delta \right)$	0.011	0.008	0.006	0.669
$E{\hat{\rho }}^{2}$	0.925	0.960	0.936	
$\hat{\Phi }$	0.858	0.930	0.931	
S/N(δ)	12.350	23.760	14.564	
S/N(Δ)	6.026	13.286	13.594	

### $p^{•}\;x\;i^{•\;}$ **and** $p^{•}\;x\;I^{•}$

**Figure 6.**
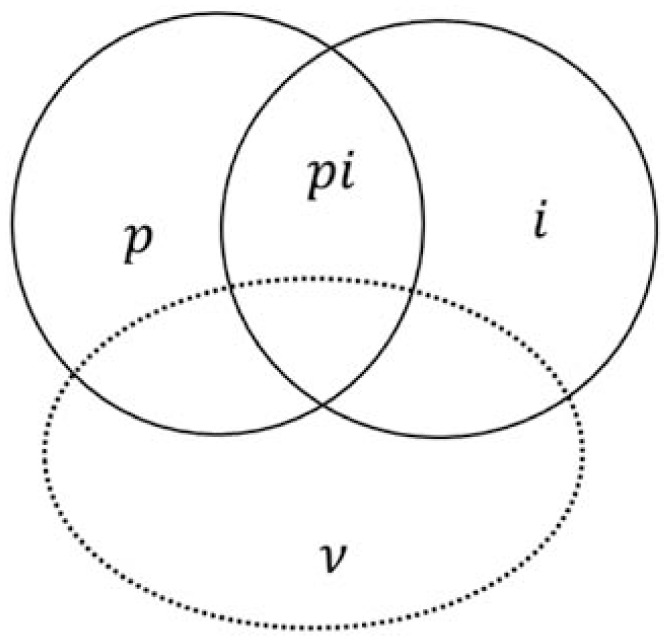
Venn diagram of $p^{•}\;x\;i^{•}$ .

The last design to consider is $p^{•}\;x\;i^{•}$ , in which persons are crossed with items. Figure 6 illustrates a Venn diagram representation. The number of persons ( $n_{p}$ ) was 1,517, and the number of items ( $n_{i})$ was 25.

Table 10 presents the variance and covariance components for both $p^{•}\;x\;i^{•}$ and $p^{•}\;x\;I^{•}$ designs. The patterns among variance components were consistent with those found in $s^{•}\;x\;i^{•}$ or $g^{•}\;x\;i^{•}$ . The universe score variances for persons were $\sigma_{\mathrm{pre}}^{2}\left(\tau \right)=0.271$ and $\sigma_{\mathrm{post}}^{2}\left(\tau \right)=0.326$ , which were the largest values among all designs. $E{\hat{\rho }}_{\mathrm{diff}}^{2}$ , ${\hat{\Phi }}_{\mathrm{diff}}$ , S/N(δ) , and S/N(Δ) are reported in Table 11. They were very large, indicating a high level of consistency and precision.

**Table 10.** Variance and Covariance Components for $p^{•}\;x\;i^{•}$ and $p^{•}\;x\;I^{•}$ .

$p^{•}\;x\;i^{•}$					$p^{•}\;x\;I^{•}$				
α	${\hat{\sigma }}_{\mathrm{pre}}^{2}$	${\hat{\sigma }}_{\mathrm{post}}^{2}$	${\hat{\sigma }}_{\mathrm{pre},\mathrm{post}}$	${\hat{\rho }}_{\mathrm{pre},\mathrm{post}}$	n	α	${\hat{\sigma }}_{\mathrm{pre}}^{2}$	${\hat{\sigma }}_{\mathrm{post}}^{2}$	${\hat{\sigma }}_{\mathrm{pre},\mathrm{post}}$
*p*	0.271	0.326	0.175	0.589		*p*	0.271	0.326	0.175
*i*	0.131	0.083	0.102	0.979	25.0	*I*	0.005	0.003	0.004
*pi*	0.610	0.525	0.153	0.270	25.0	*pI*	0.024	0.021	0.006

**Table 11.** *D* Study Results for $p^{•}\;x\;I^{•}$ for $x_{\mathrm{pre}}$ , $x_{\mathrm{post}}$ , and the Composite Score $x_{\mathrm{diff}}$ .

Statistic	$x_{\mathrm{pre}}$	$x_{\mathrm{post}}$	$x_{\mathrm{diff}}$	${\hat{\rho }}_{\mathrm{pre},\mathrm{post}}$
${\hat{\sigma }}^{2}\left(\tau \right)$	0.271	0.326	0.247	0.589
${\hat{\sigma }}^{2}\left(\delta \right)$	0.024	0.021	0.033	0.270
${\hat{\sigma }}^{2}\left(\Delta \right)$	0.030	0.024	0.034	0.380
$E{\hat{\rho }}^{2}$	0.917	0.940	0.882	
$\hat{\Phi }$	0.901	0.931	0.881	
S/N(δ)	11.113	15.551	7.458	
S/N(Δ)	9.151	13.417	7.372	

### Comparison Across Designs

There are three designs in which sites serve as the object of measurement: $\left(P^{•}:G^{•}:s^{•}\right)\;x\;I^{•}$ , $\left(P^{•}:s^{•}\right)\;x\;I^{•}$ , and $s^{•}\;x\;I^{•}$ . The corresponding coefficients for these designs are summarized in Table 12. A clear trend was observed in both the relative error variances ( ${\hat{\sigma }}^{2}\left(\delta \right)$ ) and the absolute error variances ( ${\hat{\sigma }}^{2}\left(\Delta \right)$ ), with the smallest value occurring in the $s^{•}\;x\;I^{•}$ design, and the largest in the $\left(P^{•}:G^{•}:s^{•}\right)\;x\;I^{•}$ design. The increase in ${\hat{\sigma }}^{2}\left(\delta \right)$ and ${\hat{\sigma }}^{2}\left(\Delta \right)$ accompanied by a decrease in ${\hat{\sigma }}^{2}\left(\tau \right)$ resulted in a decrease in the generalizability coefficient (i.e., $E{\hat{\rho }}^{2}$ ), dependability coefficient (i.e., $\hat{\Phi }$ ), S/N(δ) , and S/N(Δ) . These patterns illustrate how omission of person and group facets can influence the estimates of error variances and reliability-related indices.

**Table 12.** Summary of D Study Results Across Designs for Site Mean Difference Scores Where $x_{\mathrm{diff}}$ Is the Composite Score.

	$s^{•}\;x\;I^{•}$		$\left(P^{•}:s^{•}\right)\;x\;I^{•}$		$\left(P^{•}:G^{•}:s^{•}\right)\;x\;I^{•}$	
Statistic	$x_{\mathrm{diff}}$	${\hat{\rho }}_{\mathrm{pre},\mathrm{post}}$	$x_{\mathrm{diff}}$	${\hat{\rho }}_{\mathrm{pre},\mathrm{post}}$	$x_{\mathrm{diff}}$	${\hat{\rho }}_{\mathrm{pre},\mathrm{post}}$
${\hat{\sigma }}^{2}\left(\tau \right)$	0.057	0.579	0.031	0.543	0.022	0.432
${\hat{\sigma }}^{2}\left(\delta \right)$	0.003	0.412	0.012	0.574	0.036	0.609
${\hat{\sigma }}^{2}\left(\Delta \right)$	0.003	0.757	0.013	0.661	0.036	0.637
$E{\hat{\rho }}^{2}$	0.950		0.720		0.378	
$\hat{\Phi }$	0.943		0.714		0.375	
S/N(δ)	18.851		2.569		0.607	
S/N(Δ)	16.543		2.495		0.601	

As the relative error variance increased across $s^{•}\;x\;I^{•}$ , $\left(P^{•}:s^{•}\right)\;x\;I^{•}$ , and $\left(P^{•}:G^{•}:s^{•}\right)\;x\;I^{•}$ , so did the relative error correlations. As the absolute error variance increased, the absolute error correlations decreased. As summarized in Table 1, there are more variance components contributing to the relative and absolute error variances across these designs. This resulted in an increase in both error variances. Considering the variance components changed across these designs, the decrease in ${\hat{\rho }}_{\mathrm{pre},\mathrm{post}}\left(\Delta \right)$ across designs might have stemmed from the higher increase in the product of the standard deviations compared to the increase in the covariance.

In the $\left(P^{•}:G^{•}:s^{•}\right)\;x\;I^{•}$ design, the nesting indices of persons and groups were explicitly treated as facets in the universe of generalization, allowing their contributions to the error variance to be separately estimated. Similarly, in the $\left(P^{•}:s^{•}\right)\;x\;I^{•}$ design, persons were modeled as a facet, and their variability was explicitly included in the estimation of error variance. In contrast, the $s^{•}\;x\;I^{•}$ design did not specify any nesting indices, meaning that variability due to persons and groups was absorbed into the site component. Although these sources of variability still contribute to the total error variance, their impact is not directly estimated and may be underrepresented or misallocated. These differences in design specification help explain the increase in error variances, changes in error correlations, and decrease in generalizability and dependability coefficients as the model becomes more simplified and omits key facets of the data structure.

Table 13 illustrates how the coefficients vary when the object of measurement changes across persons, groups, and sites. Relative and absolute error correlations increased, and reliability-related coefficients decreased across $p^{•}\;x\;I^{•}$ , $\left(P^{•}:g^{•}\right)\;x\;I^{•}$ , and $\left(P^{•}:G^{•}:s^{•}\right)\;x\;I^{•}$ , respectively. In terms of ${\hat{\sigma }}^{2}\left(\tau \right)$ , ${\hat{\sigma }}^{2}\left(\delta \right)$ , and ${\hat{\sigma }}^{2}\left(\Delta \right)$ , there was no clear pattern across designs. This is reasonable because the D study variance components and their divisors varied depending on the specified model.

**Table 13.** Summary of D Study Results Across Different Objects of Measurement Based on the Composite Score $x_{\mathrm{diff}}$ .

	$p^{•}\;x\;I^{•}$		$\left(P^{•}:g^{•}\right)\;x\;I^{•}$		$\left(P^{•}:G^{•}:s^{•}\right)\;x\;I^{•}$	
Statistic	$x_{\mathrm{diff}}$	${\hat{\rho }}_{\mathrm{pre},\mathrm{post}}$	$x_{\mathrm{diff}}$	${\hat{\rho }}_{\mathrm{pre},\mathrm{post}}$	$x_{\mathrm{diff}}$	${\hat{\rho }}_{\mathrm{pre},\mathrm{post}}$
${\hat{\sigma }}^{2}\left(\tau \right)$	0.247	0.589	0.042	0.595	0.022	0.432
${\hat{\sigma }}^{2}\left(\delta \right)$	0.033	0.270	0.030	0.563	0.036	0.609
${\hat{\sigma }}^{2}\left(\Delta \right)$	0.034	0.380	0.031	0.605	0.036	0.637
$E{\hat{\rho }}^{2}$	0.882		0.582		0.378	
$\hat{\Phi }$	0.881		0.579		0.375	
S/N(δ)	7.458		1.390		0.607	
S/N(Δ)	7.372		1.373		0.601	

## Discussion

This study examines the reliability of difference scores obtained from multilevel pretest–posttest data. The nature of difference scores (i.e., composite scores), combined with the multilevel data structure, necessitates considering both variance and covariance components in reliability estimation. G theory, with its univariate, multivariate, and extended multivariate methodologies, provides a flexible and powerful framework for modeling reliability in such complex contexts (Brennan, 2001a; Brennan et al., 2022; Jiang et al., 2024). In this study, the following *G* study designs were investigated: $\left(p^{•}:g^{•}:s^{•}\right)\;x\;i^{•}$ , $\left(p^{•}:s^{•}\right)\;x\;i^{•}$ , $\left(p^{•}:g^{•}\right)\;x\;i^{•}$ , $g^{•}\;x\;i^{•}$ , $p^{•}\;x\;i^{•}$ , and $s^{•}\;x\;i^{•}$ using multivariate G theory. A central issue addressed in this study is how values and components of reliability coefficients, representing the magnitude and the rank order stability of difference scores, vary across designs with the same object of measurement but with neglected nested facets, and across designs with different objects of measurement.

When the focus is on decision-making regarding the relative standing of the unit of analysis, the generalizability coefficient, $E{\hat{\rho }}^{2}$ , is more appropriate to use (Brennan & Kane, 1977; Miller & Kane, 2001). In contrast, when the goal is to make a criterion-based decision, the dependability coefficient, $\hat{\Phi }$ , is more suitable. These coefficients differ from each other in terms of error variance. In this study, the values of $E{\hat{\rho }}^{2}$ and $\hat{\Phi }$ were consistently close to each other across all designs, and this pattern held for pretest, posttest, and difference scores. In all the designs, the definition of absolute error variance differs from relative error variance only by the inclusion of the item facet, and the variance associated with the item facet was relatively small. This finding holds even for all designs because neglected person and/or group facets were a nesting facet for site, not for item.

Both $E{\hat{\rho }}^{2}$ and $\hat{\Phi }$ coefficients were largest when the object of measurement was persons (i.e., students), and smallest when the object of measurement was sites. One reason behind this finding was the small sample sizes associated with groups within sites (i.e., g:s ) and persons within groups within sites (i.e., p:g:s) facets. The total number of persons was 1,517; the number of persons per group varied across groups within sites, with a harmonic mean of 8.277, and the number of groups per site varied across sites, with a harmonic mean of 2.349. If the number of persons per group per site or the number of groups per site increases, then $E{\hat{\rho }}^{2}$ and $\hat{\Phi }$ coefficients of sites will increase since these numbers were divisors in the computation of $E{\hat{\rho }}^{2}$ and $\hat{\Phi }$ coefficients. Furthermore, $E{\hat{\rho }}^{2}$ values for pretest scores were consistently smaller than those for posttest scores, a pattern that also held for $\hat{\Phi }$ . These findings are consistent with those reported by Miller and Kane (2001). One possible explanation for these patterns is the meaningful change observed between pretest and posttest scores, which may have led to increased universe score variance and, in turn, higher reliability estimates in the posttest condition.

When the object of measurement is sites, both groups and persons should be treated as facets in the universe of generalization. Sites often differ in terms of culture, background, and learning conditions. In this study, the number of groups within sites and the number of persons within groups varied substantially. Moreover, when these facets are not explicitly included in the estimation, only the site means remain, which can be used for reliability estimation. This means that the variance of the persons within groups within sites and of the groups within sites could not be completely included in the reliability estimation. In other words, the information provided by the persons and groups is lost to some extent, resulting in inflated reliability coefficients. This underscores the importance of incorporating the nested structure of the sampling design into reliability estimation. Specifically, when making decisions at the group level, person-level variance should not be ignored. Similarly, when making decisions at the site-level, group-level and person-level variance must be accounted for to avoid misleading reliability estimates.

This study decomposed the reliability of site mean difference scores and error correlations into its constituting facets via multivariate G theory. By comparing different designs with the same object of measurement, we observed that the trend in correlated errors could differ. Across $s^{•}\;x\;I^{•}$ , $\left(P^{•}:s^{•}\right)\;x\;I^{•}$ , and $\left(P^{•}:G^{•}:s^{•}\right)\;x\;I^{•}$ , the relative error correlations increased, and the absolute error correlations decreased. The absolute error includes more facets than the relative error. Absolute error correlations can decrease when the product of the absolute error standard deviations increases more rapidly than the absolute error covariance.

When difference scores are used to assess the impact of an instructional design in K-12 or learning gains in international large-scale assessments (e.g., TIMSS 2023 Longitudinal Study, Fishbein et al., 2026), or to evaluate instructional quality in higher education institutions (e.g., Buchanan et al., 2025; Rubach et al., 2025), the data have multiple levels, and the computation of difference scores should explicitly model these levels. If generalization is aimed at making decisions over sites or any unit with two levels of nesting, then the design should be $\left(P^{•}:G^{•}:s^{•}\right)\;x\;I^{•}$ . If it is over groups or any unit with one level of nesting, then the design should be $\left(P^{•}:g^{•}\right)\;x\;I^{•}$ . If it is persons, then the design should be $p^{•}\;x\;I^{•}$ .

## Supplemental Material

sj-pdf-1-epm-10.1177_00131644261451746 – Supplemental material for Reliability of Difference Scores Obtained From Nested Data Within a Multivariate Generalizability Theory Framework Supplemental material, sj-pdf-1-epm-10.1177_00131644261451746 for Reliability of Difference Scores Obtained From Nested Data Within a Multivariate Generalizability Theory Framework by Rabia Karatoprak Ersen, Won-Chan Lee and Donald B. Yarbrough in Educational and Psychological Measurement

sj-pdf-2-epm-10.1177_00131644261451746 – Supplemental material for Reliability of Difference Scores Obtained From Nested Data Within a Multivariate Generalizability Theory Framework Supplemental material, sj-pdf-2-epm-10.1177_00131644261451746 for Reliability of Difference Scores Obtained From Nested Data Within a Multivariate Generalizability Theory Framework by Rabia Karatoprak Ersen, Won-Chan Lee and Donald B. Yarbrough in Educational and Psychological Measurement
