# Supplementary material for: Reliability of Difference Scores Obtained From Nested Data Within a Multivariate Generalizability Theory Framework
Source: Educ Psychol Meas. 2026 Jul 7:00131644261451746. Online ahead of print. doi: 10.1177/00131644261451746 (PMC13346105; doi:10.1177/00131644261451746)
Supplement: sj-pdf-1-epm-10.1177_00131644261451746 – Supplemental material for Reliability of Difference Scores Obtained From Nested Data Within a Multivariate Generalizability Theory Framework [file sj-pdf-1-epm-10.1177_00131644261451746.pdf]

Multivariate G Theory Analysis

Supplement to the *Reliability of Difference Scores Obtained from Nested Data within a Multivariate Generalizability Theory Framework* Manuscript

Contents

1. Import G study variance components from the urGENOVA output 1
2. Produce G study variance and covariance matrices 3
3. Produce D study variance-covariance components 4
4. D study results for individual variables 6
5. D study results for composite 9

This supplementary material starts with importing urGENOVA outputs, and continues with presenting the analysis steps for the G study $(p^{\bullet} : g^{\bullet} : s^{\bullet}) \text{x}\, i^{\bullet}$ covariance components. Then, it demonstrates the computational procedures used to obtain the D study $(P^{\bullet} : G^{\bullet} : s^{\bullet}) \text{x}\, I^{\bullet}$ variance-covariance components, and D study coefficients for individual variables. Lastly, analysis of composite universe score and error variances, and the corresponding generalizability and dependability coefficients, and S/N ratios are shown.

The article reports the final results in summarized form, whereas this document provides the intermediate steps and outputs so that readers can follow, verify, and reproduce the reported results.

```
library(tidyverse)
library(dplyr)
```

1. Import G study variance components from the urGENOVA output

The pretest scores x_{pre}

```
d.pre <- read.fwf(file = "output/(pgs)xi_pre.in.out",
                  widths = 150, skip = 235,
                  nrows = 7,
                  blank.lines.skip = TRUE,
                  strip.white = TRUE,
                  comment.char = "-",
                  sep = "",
                  header = TRUE)
d.pre
```

```
##   Effect   df        T        SS       MS      VC
## 1      s   31 106381.1 1111.6340 35.85916 0.01287
## 2    g:s   57 107276.7  895.5795 15.71192 0.02487
```

```
## 3  p:g:s  1428 116464.6  9187.9676   6.43415 0.23430
## 4      i    24 110042.4  4772.9328 198.87220 0.12978
## 5     si   744 112329.6  1175.5830   1.58008 0.01083
## 6   gi:s  1368 114468.6  1243.4373   0.90895 0.02310
## 7 pi:g:s 34272 143418.5 19761.9069   0.57662 0.57662
```

The posttest scores x_{post}

```
d.post <- read.fwf(file = "output/(pgs)xi_post.in.out",
                 widths = 150, skip = 235,
                 nrows = 7,
                 blank.lines.skip = TRUE,
                 strip.white = TRUE,
                 comment.char = "-",
                 sep = "",
                 header = TRUE)
d.post
```

```
##   Effect    df        T         SS        MS      VC
## 1      s    31 154598.7  1784.6479  57.56929 0.02431
## 2    g:s    57 155810.1  1211.4554  21.25360 0.03866
## 3  p:g:s  1428 165977.6 10167.4590   7.12007 0.26517
## 4      i    24 155864.5  3050.4975 127.10406 0.08231
## 5     si   744 158928.4  1279.2486   1.71942 0.01888
## 6   gi:s  1368 161127.6   987.7151   0.72201 0.01607
## 7 pi:g:s 34272 188116.0 16820.9389   0.49081 0.49081
```

The sum scores $x_{sum} = x_{post} + x_{pre}$

```
d.sum <- read.fwf(file = "output/(pgs)xi_sum.in.out",
                 widths = 150, skip = 235,
                 nrows = 7,
                 blank.lines.skip = TRUE,
                 strip.white = TRUE,
                 comment.char = "-",
                 sep = "",
                 header = TRUE)
d.sum
```

```
##   Effect    df        T        SS        MS      VC
## 1      s    31 516190.1  4440.231 143.23325 0.05245
## 2    g:s    57 519619.3  3429.215  60.16167 0.10614
## 3  p:g:s  1428 549839.4 30220.133  21.16256 0.79315
## 4      i    24 527024.6 15274.752 636.44799 0.41524
## 5     si   744 535175.8  3711.012   4.98792 0.05162
## 6   gi:s  1368 541563.8  2958.735   2.16282 0.05763
## 7 pi:g:s 34272 617494.0 45710.101   1.33374 1.33374
```

```r
(var_comp <- data.frame(effect =  d.pre[,1],
                        var_sum=d.sum[,6],
                        var_pre =d.pre[, 6],
                        var_post=d.post[,6]))
```

```
##    effect var_sum var_pre var_post
## 1       s 0.05245 0.01287  0.02431
## 2     g:s 0.10614 0.02487  0.03866
## 3   p:g:s 0.79315 0.23430  0.26517
## 4       i 0.41524 0.12978  0.08231
## 5      si 0.05162 0.01083  0.01888
## 6    gi:s 0.05763 0.02310  0.01607
## 7  pi:g:s 1.33374 0.57662  0.49081
```

2. Produce G study variance and covariance matrices

Derive covariance component for each effect

The covariance component $\sigma_{pre,post}(\alpha)$ for each effect was derived using the following equation:

$$\sigma_{pre,post}(\alpha) = \frac{\sigma^2_{sum}(\alpha) - \sigma^2_{pre}(\alpha) + \sigma^2_{post}(\alpha)}{2}$$

.

```r
cov_comp <- function(pre, post, sum) {

    #var(sum) = var(pre) + var(post) + 2. cov(pre, post)
    #cov(pre, post) = [var(sum) - var(pre) - var(post)] / 2
    cov_sum = (sum - pre - post) / 2
    return(cov_sum)
}
```

```r
(var_covar_comp <- var_comp %>%
     mutate(cov_prepost = cov_comp(sum = var_sum, pre = var_pre, post = var_post)))
```

```
##    effect var_sum var_pre var_post cov_prepost
## 1       s 0.05245 0.01287  0.02431    0.007635
## 2     g:s 0.10614 0.02487  0.03866    0.021305
## 3   p:g:s 0.79315 0.23430  0.26517    0.146840
## 4       i 0.41524 0.12978  0.08231    0.101575
## 5      si 0.05162 0.01083  0.01888    0.010955
## 6    gi:s 0.05763 0.02310  0.01607    0.009230
## 7  pi:g:s 1.33374 0.57662  0.49081    0.133155
```

Compute disattenuated correlations

```r
dis_corr <- function(var1, var2, cov) {

    corr = cov / sqrt(var1 * var2)
    return(corr)
}
```

```r
gstudy <- var_covar_comp %>%
    mutate(dis_corr = dis_corr(var1=var_pre, var2 = var_post, cov = cov_prepost)) %>%
    select(-var_sum)
gstudy
```

```
##   effect var_pre var_post cov_prepost  dis_corr
## 1      s 0.01287  0.02431    0.007635 0.4316456
## 2    g:s 0.02487  0.03866    0.021305 0.6870886
## 3  p:g:s 0.23430  0.26517    0.146840 0.5891095
## 4      i 0.12978  0.08231    0.101575 0.9827808
## 5     si 0.01083  0.01888    0.010955 0.7661206
## 6   gi:s 0.02310  0.01607    0.009230 0.4790576
## 7 pi:g:s 0.57662  0.49081    0.133155 0.2502972
```

Variance-covariance components for $(p^{\bullet} : g^{\bullet} : s^{\bullet})\, \mathbf{x}\, i^{\bullet}$

The following output reproduces G study the variance–covariance components reported in Equation 5-11.

```r
bind_cols(effect = gstudy$effect, round(gstudy[,-1], 5))
```

```
##   effect var_pre var_post cov_prepost dis_corr
## 1      s 0.01287  0.02431     0.00763  0.43165
## 2    g:s 0.02487  0.03866     0.02130  0.68709
## 3  p:g:s 0.23430  0.26517     0.14684  0.58911
## 4      i 0.12978  0.08231     0.10158  0.98278
## 5     si 0.01083  0.01888     0.01096  0.76612
## 6   gi:s 0.02310  0.01607     0.00923  0.47906
## 7 pi:g:s 0.57662  0.49081     0.13315  0.25030
```

3. Produce D study variance-covariance components

Sample size statistics

The following sample sizes were used in the computations. Harmonic means are taken over the sample sizes to estimate divisor in computing D study variance and covariance components. For their computation, please see the article.

```r
n_pgs <- 8.276944 #the number of persons within each group and site
n_gs <- 2.348624 #the number of groups within each site
n_i <- 25
```

Diagonal divisor for each D study effect

```r
n_Gs <- n_gs # G:s
n_PGs <- n_pgs # P:G:s
n_I <- n_i # I
n_sI <- n_i # sI
n_GIs <- n_gs * n_i # GI:s
n_PIGs <- n_pgs * n_gs * n_i # PI:G:s
```

Compute D study variance-covariance components

```
s_pre <- gstudy$var_pre[1]
s_post <- gstudy$var_post[1]
s_cov <- gstudy$cov_prepost[1]
s_corr <- dis_corr(var1=s_pre, var2=s_post, cov = s_cov)
```

s

```
#G:s
Gs_pre <- gstudy$var_pre[2]/ n_Gs
Gs_post <- gstudy$var_post[2]/ n_Gs
Gs_cov <- gstudy$cov_prepost[2]/ n_Gs
Gs_corr <- dis_corr(var1= Gs_pre, var2= Gs_post, cov = Gs_cov)
```

G:s

```
#P:G:s
PGs_pre <- gstudy$var_pre[3]/n_PGs
PGs_post <- gstudy$var_post[3]/n_PGs
PGs_cov <- gstudy$cov_prepost[3]/n_PGs
PGs_corr <- dis_corr(var1= PGs_pre, var2= PGs_post, cov = PGs_cov)
```

P:G:s

```
#I
I_pre <- gstudy$var_pre[4]/n_I
I_post <- gstudy$var_post[4]/n_I
I_cov <- gstudy$cov_prepost[4]/n_I
I_corr <- dis_corr(var1= I_pre, var2= I_post, cov = I_cov)
```

I

```
#sI
sI_pre <- gstudy$var_pre[5]/n_sI
sI_post <- gstudy$var_post[5]/n_sI
sI_cov <- gstudy$cov_prepost[5]/n_sI
sI_corr <- dis_corr(var1= sI_pre, var2= sI_post, cov = sI_cov)
```

sI

```
#GI:s
GIs_pre <- gstudy$var_pre[6]/n_GIs
GIs_post <- gstudy$var_post[6]/n_GIs
GIs_cov <- gstudy$cov_prepost[6]/n_GIs
GIs_corr <- dis_corr(var1= GIs_pre, var2= GIs_post, cov = GIs_cov)
```

GI:s

```
#PI:G:s
PIGs_pre <- gstudy$var_pre[7]/n_PIGs
PIGs_post <- gstudy$var_post[7]/n_PIGs
PIGs_cov <- gstudy$cov_prepost[7]/n_PIGs
PIGs_corr <- dis_corr(var1= PIGs_pre, var2= PIGs_post, cov = PIGs_cov)
```

PI:G:s

Variance-covariance components for $(P^{\bullet} : G^{\bullet} : s^{\bullet})\,\mathrm{x}\,I^{\bullet}$

```
dstudy <- data.frame(divisor = c(NA, n_Gs, n_PGs, n_I, n_sI, n_GIs, n_PIGs),
                     effect = c("s", "G:s", "P:G:s", "I", "sI", "GI:s", "PI:G:s"),
                     var_pre=c(s_pre, Gs_pre, PGs_pre, I_pre, sI_pre, GIs_pre, PIGs_pre),
                     var_post=c(s_post, Gs_post, PGs_post, I_post, sI_post, GIs_post, PIGs_post),
                     cov_prepost=c(s_cov, Gs_cov, PGs_cov, I_cov, sI_cov, GIs_cov, PIGs_cov),
                     corr_prepost=c(s_corr, Gs_corr, PGs_corr, I_corr, sI_corr, GIs_corr, PIGs_corr)
                     )
```

The following output reproduces the variance–covariance components for $(P^{\bullet} : G^{\bullet} : s^{\bullet})\,\mathrm{x}\,I^{\bullet}$ reported in Equation 12-17, after rounding to the same number of decimal places.

```
dstudy
```

```
##      divisor effect      var_pre     var_post  cov_prepost corr_prepost
## 1         NA      s 0.0128700000 0.0243100000 0.0076350000    0.4316456
## 2   2.348624    G:s 0.0105891790 0.0164607021 0.0090712690    0.6870886
## 3   8.276944  P:G:s 0.0283075493 0.0320371867 0.0177408473    0.5891095
## 4  25.000000      I 0.0051912000 0.0032924000 0.0040630000    0.9827808
## 5  25.000000     sI 0.0004332000 0.0007552000 0.0004382000    0.7661206
## 6  58.715600   GI:s 0.0003934219 0.0002736922 0.0001571984    0.4790576
## 7 485.985733 PI:G:s 0.0011864957 0.0010099268 0.0002739895    0.2502972
```

4. D study results for individual variables

Compute the universe score and error variance-covariance matrices

The values reported in the following chunks correspond to the entries of the universe score, relative error, and absolute error variance-covariance matrices. In the article, these results are presented in matrix form.

The universe score variance-covariance matrix was obtained as

$$\Sigma_\tau = \Sigma_s = \begin{bmatrix} \sigma^2_{pre}(s) & \sigma_{pre,post}(s) \\ \sigma_{pre,post}(s) & \sigma^2_{post}(s) \end{bmatrix}$$

.

```
univ_pre <- dstudy[ dstudy$effect == "s", "var_pre"]
univ_post<- dstudy[ dstudy$effect == "s", "var_post"]
univ_cov<- dstudy[ dstudy$effect == "s", "cov_prepost"]
univ_corr <- dis_corr(var1=univ_pre, var2=univ_post, cov = univ_cov)
```

The relative error variance-covariance matrix was calculated using the D study variance-covariance matrices as

$$\Sigma_\delta = \Sigma_{G:s} + \Sigma_{P:G:s} + \Sigma_{sI} + \Sigma_{GI:s} + \Sigma_{PI:G:s}.$$

```
dstudy[!(dstudy$effect == "I"| dstudy$effect == "s"), "effect"]
```

```
## [1] "G:s"    "P:G:s"  "sI"     "GI:s"   "PI:G:s"
```

```
rel_pre <- sum(dstudy[!(dstudy$effect == "I"| dstudy$effect == "s"), "var_pre"])
rel_post <- sum(dstudy[!(dstudy$effect == "I"| dstudy$effect == "s"), "var_post"])
rel_cov <- sum(dstudy[!(dstudy$effect == "I"| dstudy$effect == "s"), "cov_prepost"])
rel_corr <- dis_corr(var1=rel_pre, var2=rel_post, cov = rel_cov)
```

The absolute error variance-covariance matrix was obtained as

$$\Sigma_\Delta = \Sigma_{G:s} + \Sigma_{P:G:s} + \Sigma_I + \Sigma_{sI} + \Sigma_{GI:s} + \Sigma_{PI:G:s}.$$

```
dstudy[!(dstudy$effect == "s"), "effect"]
```

```
## [1] "G:s"    "P:G:s"  "I"      "sI"     "GI:s"   "PI:G:s"
```

```
abs_pre <- sum(dstudy[!(dstudy$effect == "s"), "var_pre"])
abs_post <- sum(dstudy[!(dstudy$effect == "s"), "var_post"])
abs_cov <- sum(dstudy[!(dstudy$effect == "s"), "cov_prepost"])
abs_corr <- dis_corr(var1 =abs_pre, var2 = abs_post, cov = abs_cov)
```

```
df <- data.frame(effect = c("universe", "rel error", "abs error"),
                 var_pre= c(univ_pre, rel_pre, abs_pre),
                 var_post= c(univ_post, rel_post, abs_post),
                 cov_prepost= c(univ_cov, rel_cov, abs_cov),
                 corr_prepost= c(univ_corr, rel_corr, abs_corr))
```

The following output reproduces the results for $(P^\bullet : G^\bullet : s^\bullet)\,\text{x}\,I^\bullet$ reported in Equation 18, 21, and 22, after rounding to the same number of decimal places.

```r
df
```

```
##      effect    var_pre   var_post cov_prepost corr_prepost
## 1  universe 0.01287000 0.02431000   0.0076350    0.4316456
## 2 rel error 0.04090985 0.05053671   0.0276815    0.6087967
## 3 abs error 0.04610105 0.05382911   0.0317445    0.6372423
```

Compute reliability coefficients:

```r
relb.coef <- function(univ, err){

    relb <- univ / (univ + err)
    return(relb)
}
```

Generalizability Coefficient

$$\mathbf{E}\rho^2 = \frac{\sigma^2(\tau)}{\sigma^2(\tau) + \sigma^2(\delta)}.$$

```r
gen_pre <- relb.coef(univ = univ_pre, err = rel_pre)
gen_post <- relb.coef(univ = univ_post, err = rel_post)
```

Dependability Coefficient Dependability coefficient is:

$$\Phi = \frac{\sigma^2(\tau)}{\sigma^2(\tau) + \sigma^2(\Delta)}.$$

```r
dep_pre <- relb.coef(univ = univ_pre, err = abs_pre)
dep_post <- relb.coef(univ = univ_post, err = abs_post)
```

```r
sn.ratio <- function(univ, err){

    sn <- univ / err
    return(sn)
}
```

S/N Ratio The signal-noise ratio (S/N) represents the ratio of universe score variance to error variance. When error variance is relative error:

$$S/N(\delta) = \frac{\sigma^2(\tau)}{\sigma^2(\delta)}.$$

```r
sn.rel_pre <- sn.ratio(univ_pre, rel_pre)
sn.rel_post <- sn.ratio(univ_post, rel_post)
```

When error variance is absolute error:

$$S/N(\Delta) = \frac{\sigma^2(\tau)}{\sigma^2(\Delta)}.$$

```r
sn.abs_pre <- sn.ratio(univ_pre, abs_pre)
sn.abs_post <- sn.ratio(univ_post, abs_post)
```

5. D study results for composite

The focus of this study is difference scores. Multivariate G theory treats difference scores as composite scores. In this study, the typical definition of difference scores is used to define composite scores such that

$$x_{diff} = x_{post} - x_{pre}$$

.

The fixed multivariate variable (ν) had two levels: pretest and posttest. Thus, the weights (w) for pretest and posttest were -1 and +1, respectively. Then the variance of the composite score is

$$\sigma^2_{diff}(\alpha) = \sigma^2_{post}(\alpha) + \sigma^2_{pre}(\alpha) - 2\sigma_{pre,post}(\alpha)$$

. This equation is used to compute composite universe score variance, $\sigma^2_{diff}(\tau)$, composite error variances, $\sigma^2_{diff}(\delta)$ and $\sigma^2_{diff}(\Delta)$.

```r
composite <- function(pre, post, covar) {

    #var(dif) = var(pre) + var(post) - 2. cov(pre, post)
    composite = (pre + post - 2*covar)
    return(composite)
}
```

Composite Universe Score Variance

```r
univ_comp <- composite(pre = univ_pre, post=univ_post, covar = univ_cov)
```

Composite Relative Error Variance

```r
rel_comp <- composite(pre = rel_pre, post = rel_post, covar = rel_cov)
```

Composite Absolute Error Variance

```r
abs_comp <- composite(pre = abs_pre, post = abs_post, covar = abs_cov)
```

Composite Generalizability Coefficient

```r
gen_comp <- relb.coef(univ = univ_comp, err = rel_comp)
```

Composite Dependability Coefficient

```r
dep_comp <- relb.coef(univ = univ_comp, err = abs_comp)
```

S/N-Rel Ratio

```r
sn.rel_comp <- sn.ratio(univ_comp, rel_comp)
```

S/N-Abs Ratio

```r
sn.abs_comp <- sn.ratio(univ_comp, abs_comp)
```

```r
composite <- data.frame(
    label = c("Universe Score Variance", "Relative Error Variance", "Absolute Error Variance",
              "Gen Coefficient", "Phi Coefficient", "S/N-Relative", "S/N-Absolute"),
    Pre = c(univ_pre, rel_pre, abs_pre, gen_pre, dep_pre, sn.rel_pre, sn.abs_pre),
    Post = c(univ_post, rel_post, abs_post, gen_post, dep_post, sn.rel_post, sn.abs_post),
    Composite = c(univ_comp, rel_comp, abs_comp, gen_comp, dep_comp, sn.rel_comp, sn.abs_comp)
                        )
```

The following output reproduces the D study results for x_{pre}, x_{post}, and the composite score x_{diff} reported in Equation 27-37, after rounding to the same number of decimal places.

```r
composite
```

```
##                     label        Pre       Post  Composite
## 1 Universe Score Variance 0.01287000 0.02431000 0.02191000
## 2 Relative Error Variance 0.04090985 0.05053671 0.03608355
## 3 Absolute Error Variance 0.04610105 0.05382911 0.03644115
## 4         Gen Coefficient 0.23930898 0.32479719 0.37780067
## 5         Phi Coefficient 0.21824270 0.31111182 0.37548535
## 6            S/N-Relative 0.31459419 0.48103648 0.60720198
## 7            S/N-Absolute 0.27916937 0.45161440 0.60124346
```
