# Supplementary material for: Reliability of Difference Scores Obtained From Nested Data Within a Multivariate Generalizability Theory Framework
Source: Educ Psychol Meas. 2026 Jul 7:00131644261451746. Online ahead of print. doi: 10.1177/00131644261451746 (PMC13346105; doi:10.1177/00131644261451746)
Supplement: sj-pdf-2-epm-10.1177_00131644261451746 – Supplemental material for Reliability of Difference Scores Obtained From Nested Data Within a Multivariate Generalizability Theory Framework [file sj-pdf-2-epm-10.1177_00131644261451746.pdf]

Univariate G Theory Analysis

Supplement to the *Reliability of Difference Scores Obtained from Nested Data within a Multivariate Generalizability Theory Framework* Manuscript

Contents

1. Import G study variance components from the urGENOVA output 1
2. Produce D study variance-covariance components 2
3. D study results 3

This supplementary material presents the Univariate G theory analysis steps for the D study $(P : G : s) \, \mathrm{x} \, I$ variance components, universe score and error variances, and the corresponding generalizability and dependability coefficients. The article reports and discusses the Multivariate results, whereas this document provides the univariate analysis as a supplementary material.

```
library(tidyverse)
library(dplyr)
```

1. Import G study variance components from the urGENOVA output

Variance components for posttest scores x_{post} minus pretest scores x_{pre}

```
d.dif <- read.fwf(file = "output/(pgs)xi_dif.in.out",
                  widths = 150, skip = 235,
                  nrows = 7,
                  blank.lines.skip = TRUE,
                  strip.white = TRUE,
                  comment.char = "-",
                  sep = "",
                  header = TRUE)
d.dif
```

```
##   Effect    df         T         SS       MS      VC
## 1      s    31  5769.505  1352.3331 43.62365 0.02190
## 2    g:s    57  6554.360   784.8548 13.76938 0.02093
## 3  p:g:s  1428 15045.080  8490.7201  5.94588 0.20579
## 4      i    24  4789.281   372.1088 15.50453 0.00894
## 5     si   744  7340.265  1198.6514  1.61109 0.00779
## 6   gi:s  1368  9628.690  1503.5694  1.09910 0.02072
## 7 pi:g:s 34272 45575.000 27455.5904  0.80111 0.80111
```

```
gstudy <- data.frame(effect =  d.dif[, "Effect"], var_post_pre=d.dif[, "VC"])
```

2. Produce D study variance-covariance components

Sample size statistics

The following sample sizes were used in the computations. Harmonic means are taken over the sample sizes to estimate divisor in computing D study variance and covariance components. For their computation, please see the article.

```
n_pgs <- 8.276944 #the number of persons within each group and site
n_gs <- 2.348624 #the number of groups within each site
n_i <- 25
```

Diagonal divisor for each D study effect

```
n_Gs <- n_gs # G:s
n_PGs <- n_pgs # P:G:s
n_I <- n_i # I
n_sI <- n_i # sI
n_GIs <- n_gs * n_i # GI:s
n_PIGs <- n_pgs * n_gs * n_i # PI:G:s
```

Compute D study variance components

```
#s
s <- gstudy$var_post_pre[1]
#G:s
Gs <- gstudy$var_post_pre[2]/ n_Gs
#P:G:s
PGs <- gstudy$var_post_pre[3]/n_PGs
#I
I <- gstudy$var_post_pre[4]/n_I
#sI
sI <- gstudy$var_post_pre[5]/n_sI
#GI:s
GIs <- gstudy$var_post_pre[6]/n_GIs
#PI:G:s
PIGs <- gstudy$var_post_pre[7]/n_PIGs
```

Variance components for $(P : G : s)$ x I

```
dstudy <- data.frame(divisor = c(NA, n_Gs, n_PGs, n_I, n_sI, n_GIs, n_PIGs),
                     effect = c("s", "G:s", "P:G:s", "I", "sI", "GI:s", "PI:G:s"),
                     var=c(s, Gs, PGs, I, sI, GIs, PIGs)
                     )
```

The following output reproduces the variance components for $(P:G:s)\,\mathrm{x}\,I$.

```
dstudy
```

```
##      divisor effect          var
## 1         NA      s 0.0219000000
## 2   2.348624    G:s 0.0089116010
## 3   8.276944  P:G:s 0.0248630412
## 4  25.000000      I 0.0003576000
## 5  25.000000     sI 0.0003116000
## 6  58.715600   GI:s 0.0003528875
## 7 485.985733 PI:G:s 0.0016484229
```

3. D study results

Compute the universe score and error variance

The values reported in the following chunks correspond to the entries of the universe score, relative error, and absolute error variance.

The universe score variance is $\sigma^2_\tau = \sigma^2_s$.

```
universe <- dstudy[ dstudy$effect == "s", "var"]
```

The relative error variance is calculated using the D study variance components as

$$\sigma^2(\delta) = \sigma^2(G:s) + \sigma^2(P:G:s) + \sigma^2(sI) + \sigma^2(GI:s) + \sigma^2(PI:G:s)$$

```
dstudy[!(dstudy$effect == "I"| dstudy$effect == "s"), "effect"]
```

```
## [1] "G:s"    "P:G:s"  "sI"     "GI:s"   "PI:G:s"
```

```
rel_error <- sum(dstudy[!(dstudy$effect == "I"| dstudy$effect == "s"), "var"])
```

The absolute error variance is obtained as

$$\sigma^2(\Delta) = \sigma^2(G:s) + \sigma^2(P:G:s) + \sigma^2(I) + \sigma^2(sI) + \sigma^2(GI:s) + \sigma^2(PI:G:s)$$

```
dstudy[!(dstudy$effect == "s"), "effect"]
```

```
## [1] "G:s"    "P:G:s"  "I"      "sI"     "GI:s"   "PI:G:s"
```

```
abs_error <- sum(dstudy[!(dstudy$effect == "s"), "var"])
```

```
df <- data.frame(effect = c("universe", "rel error", "abs error"),
                 var= c(universe, rel_error, abs_error))
df
```

```
##      effect        var
## 1  universe 0.02190000
## 2 rel error 0.03608755
## 3 abs error 0.03644515
```

Compute generalizability and dependability coefficients:

```r
relb.coef <- function(univ, err){

    relb <- univ / (univ + err)
    return(relb)
}
```

Generalizability Coefficient

$$\mathbf{E}\rho^2 = \frac{\sigma^2(\tau)}{\sigma^2(\tau) + \sigma^2(\delta)}$$

```r
gen_coef <- relb.coef(univ = universe, err = rel_error)
```

Dependability Coefficient Dependability coefficient is:

$$\Phi = \frac{\sigma^2(\tau)}{\sigma^2(\tau) + \sigma^2(\Delta)}$$

```r
dep_coef <- relb.coef(univ = universe, err = abs_error)
```

S/N Ratio

The signal-noise ratio (S/N) represents the ratio of universe score variance to error variance.

```r
sn.ratio <- function(univ, err){

    sn <- univ / err
    return(sn)
}
```

S/N-Rel Ratio When error variance is relative error:

$$S/N(\delta) = \frac{\sigma^2(\tau)}{\sigma^2(\delta)}.$$

```r
sn_rel <- sn.ratio(universe, rel_error)
```

S/N-Abs Ratio When error variance is absolute error:

$$S/N(\Delta) = \frac{\sigma^2(\tau)}{\sigma^2(\Delta)}.$$

```r
sn_abs <- sn.ratio(universe, abs_error)
```

```r
dstudy_coef <- data.frame(
    label = c("Universe Score Variance", "Relative Error Variance", "Absolute Error Variance",
              "Gen Coefficient", "Phi Coefficient", "S/N-Relative", "S/N-Absolute"),
    value = c(universe, rel_error, abs_error, gen_coef, dep_coef, sn_rel, sn_abs))
```

The following output reproduces the D study results for x_{post} minus x_{pre}.

```
dstudy_coef
```

```
##                      label      value
## 1 Universe Score Variance 0.02190000
## 2 Relative Error Variance 0.03608755
## 3 Absolute Error Variance 0.03644515
## 4         Gen Coefficient 0.37766726
## 5         Phi Coefficient 0.37535252
## 6            S/N-Relative 0.60685745
## 7            S/N-Absolute 0.60090296
```
